# Applying Absolute Free Energy Perturbation Molecular Dynamics to Diffusively Binding Ligands

**DOI:** 10.1021/acs.jctc.5c00121

**Published:** 2025-04-07

**Authors:** Xavier E. Laracuente, Bryan M. Delfing, Xingyu Luo, Audrey Olson, William Jeffries, Steven R. Bowers, Kenneth W. Foreman, Kyung Hyeon Lee, Mikell Paige, Kylene Kehn-Hall, Christopher Lockhart, Dmitri K. Klimov

**Affiliations:** † School of Systems Biology, George Mason University, Manassas, Virginia 20110, United States; ‡ Department of Chemistry and Biochemistry, George Mason University, Fairfax, Virginia 22030, United States; § Center for Molecular Engineering, George Mason University, Manassas, Virginia 20110, United States; ∥ Department of Biomedical Sciences and Pathobiology, Virginia-Maryland College of Veterinary Medicine, Virginia Polytechnic Institute and State University, Blacksburg, Virginia 24061, United States; ⊥ Center for Emerging, Zoonotic, and Arthropod-borne Pathogens, Virginia Polytechnic Institute and State University, Blacksburg, Virginia 24061, United States

## Abstract

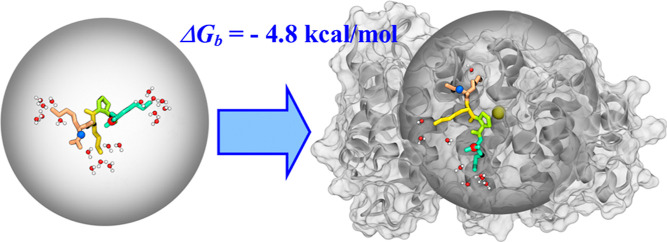

We have developed and tested an absolute free energy perturbation (FEP) protocol, which combines all-atom molecular dynamics, replica exchange with solute tempering (REST) enhanced sampling, and a spherical harmonic restraint applied to a ligand. Our objective was to compute the binding free energy together with the underlying binding mechanism for a ligand, which binds diffusively to a protein. Such ligands represent nearly impossible targets for traditional FEP simulations. To test our FEP/REST protocol, we selected a conserved motif peptide KKPK termed minNLS from the nuclear localization signal sequence of the Venezuelan equine encephalitis virus capsid protein. This peptide fragment binds diffusively to importin-α transport protein without forming well-defined poses. Our FEP/REST simulations with a spherical restraint provided a converged estimate of minNLS binding free energy. We found that minNLS binds with moderate affinity to importin-α utilizing an unusual, purely entropic mechanism in which binding free energy is determined by favorable entropic gain. For this cationic minNLS peptide, a favorable binding entropic gain is primarily associated with the release of water from the solvation shells of charged amino acids. We demonstrated that FEP/REST simulations sample the KKPK bound ensemble well, allowing us to characterize the distribution of bound structures, binding interactions, and locations on the importin-α surface. Analysis of experimental studies offered support to our rationale behind the KKPK entropic binding mechanism.

## 1 Introduction

Estimation of the binding affinities of ligands, including small peptides, to protein targets is a critical step in modern drug development. Although high-throughput screening of ligands based on automated experimental testing of their affinities offers rapid input for drug discovery, its costs can be prohibitive.^1^ A much cheaper complementary approach utilizes virtual screening of ligands using approximate but rapid scoring functions or employing artificial intelligence tools, provided they have been trained on experimental data for similar compounds.^2,3^ Consequently, it may be challenging to extend virtual screening to novel ligands with little similarity to those already studied experimentally. In such cases, one can evaluate binding of ligand to protein targets resorting to more traditional, although no less challenging, physics-based methods, which generate the conformational ensembles of binding molecules through molecular dynamics (MD) simulations.^4,5^ The principal task in these bottom-up simulation methods is the computation of the binding free energy Δ*G*_b_ of a ligand to a protein.^6^ Knowledge of Δ*G*_b_ can guide drug development or assess protein−protein or protein–nucleic acid interactions. Usually, MD approaches to Δ*G*_b_ computation use all-atom explicit solvent models and the free energy perturbation (FEP), a theoretically rigorous method for computing Δ*G*_b_ originating from Zwanzig’s formalism.^7^ The first FEP simulations have been performed almost 50 years ago,^8^ and over the elapsed years the improvements in biomolecular force fields and sampling algorithms as well as rise in computational power have made FEP a viable tool for drug development.^9,10^ In principle, FEP simulations can either estimate the absolute free energy of binding of a ligand Δ*G*_b_ or determine the changes in binding free energy ΔΔ*G*_b_ caused by modifications in the ligand chemical structure.^9,11^ Important advantages of FEP simulations are that they (i) are transferable between ligands, (ii) do not require prior experimental information, and (iii) provide bottom-up information about the molecular factors impacting Δ*G*_b_ or ΔΔ*G*_b_.

Traditional protocols for absolute FEP, which compute Δ*G*_b_ for a ligand binding to a protein, are relatively straightforward. Using the double decoupling approach,^12−14^ one needs to design a thermodynamic cycle, which (i) restrains a ligand in the unbound state, (ii) alchemically decouples the unbound ligand transferring it from the unbound “coupled” state to vacuum “uncoupled” state, (iii) alchemically recouples a ligand with the protein and solvent in the bound state, and (iv) releases the restraints.^15^ One of the main difficulties in FEP implementation is sampling convergence, which has received relatively scarce attention in the literature.^16,17^ As long as a ligand adopts a conformationally restricted, well-defined bound state, traditional FEP relying on continuous MD simulations without enhanced sampling may suffice. The convergence of such FEP simulations is facilitated by imposing the restraints proposed by Karplus and co-workers, which restrict the pose and conformation of a ligand to those observed experimentally.^18^ However, FEP convergence becomes problematic if a ligand exhibits multiple binding states. Although Mobley and Klimovich have designed a protocol for handling multiple distinct ligand states,^19^ the convergence issues become exacerbated if a ligand binds to a protein diffusively without forming well-defined poses. In this case, the Karplus restraints cannot be applied because the ligand must sample a continuous multitude of bound states. Under these conditions, a poor, far from exhaustive sampling of states collected from alchemical windows during traditional FEP will lead to failures in its convergence,^17^ rendering the evaluation of Δ*G*_b_ unreliable. This problem may be even further compounded if a protein, to which a ligand is bound, undergoes conformational restructuring. To address convergence issues, the FEP approach was combined with replica exchange with the solute tempering (REST) algorithm.^20,21^ REST enhances the conformational sampling by tempering a ligand and protein binding site, which are affected by alchemical transformation. FEP/REST simulations utilize multiple conditions with unique combinations of coupling parameter λ, which governs alchemical transformation, and temperature *T*, which governs sampling. Due to limited computational resources, FEP/REST conditions are typically arranged in one dimension, in which the terminal conditions with λ = 0 and 1 feature physiological temperature *T*. For the intermediate conditions within the intervals 0 < λ ≤ 0.5 and 0.5 < λ < 1, the temperature exponentially increases or decreases, respectively. Although FEP/REST requires parallel simulations of multiple replicas that significantly increases the computational costs over traditional FEP, the method can reduce the errors in free energy evaluation down to about 1 kcal/mol.^22,23^

However, by itself, FEP/REST does not fully resolve the problem of accurate computation of Δ*G*_b_ for ligands binding diffusively via multiple overlapping distributed binding locations. Without restraints, such a ligand can unbind or drift out of the binding region, compromising sampling of relevant bound states. To address this issue, we explore in this paper the application of a single harmonic spherical restraint, which encompasses the distributed binding region on a protein. Specifically, within this region, the restraint is inactive, but turns on if a ligand ventures beyond the boundary of the distributed binding region. The advantage of this approach is that apart from a soft spherical boundary, no restraints on ligand conformations or poses are introduced. The challenge arising with the proposed spherical restraint is a vast conformational binding space available for a ligand, which must be adequately sampled. Thus, the novelty of our approach is twofold. First, we demonstrate the utility of “laissez-faire” restraint in the context of FEP simulations. Second, we show that absolute FEP simulations can be successfully applied to the ligand binding to a protein diffusively via multiple distributed binding sites.

As a case study of diffusively binding ligand, we consider the nuclear localization signal (NLS) peptide from Venezuelan equine encephalitis virus (VEEV) capsid protein.^24^ The N-terminus of the VEEV capsid protein harbors an NLS sequence, which binds to the nuclear transport protein importin-α (impα), which in turn forms a complex with its partner protein importin-β. VEEV capsid also recruits a nuclear export protein CRM1, and the resulting tetrameric complex clogs the nuclear channel interfering with host nucleocytoplasmic traffic.^25^ Formation of this tetrameric complex, including the binding of VEEV NLS to impα, appears as a key step in VEEV pathogenesis.^25,26^ The peptide KKPK termed minNLS, which is used in this paper, is taken from VEEV NLS sequence A_5_KKPKKE_11_ and matches the classic monopartite consensus K-K/R-X-K/R motif.^27^ The X-ray structure of the complex formed by the extended VEEV NLS sequence E_1_GPSAKKPKKEA_12_ with the mouse impα protein (the PDB code 3VE6) is available.^28^ Within this structure, the KKPK fragment adopts a pose with generally low *B*-factors tightly bound to the impα major NLS binding site. Strikingly, our recent MD simulations have showed that when extracted from the NLS sequence the minNLS KKPK fragment fails to reproduce the crystallographic native binding structure, instead forming a manifold of non-native loosely bound poses within the impα major NLS binding site.^29^ We found that only the extension of the minNLS sequence by two additional C-terminal amino acids secures the native binding of the resulting coreNLS fragment KKPKKE. Previous experimental studies have estimated the typical free energy of binding of a NLS sequence, e.g., from SV40 virus, to impα to be around −10 kcal/mol.^30^ A recent report found that some NLS sequences, for example, from chloride intracellular channel proteins, may have lower affinities of about −5 kcal/mol.^31^ Because KKPK binds via diffusive mechanism radically different from that exhibited by longer NLS sequences, such as coreNLS fragment KKPKKE,^29^ it is interesting to evaluate its binding affinity. Consequently, in this paper, we use the absolute FEP/REST simulations with novel spherical restraint to compute the standard free energy of binding Δ*G*_b_° of minNLS KKPK peptide to impα. We analyze the convergence of these simulations, the binding mechanism, and the distributed bound conformational ensemble sampled by KKPK. Because our FEP/REST protocol is novel, we test its sampling of KKPK bound ensemble against prior exhaustive REST simulations.^29^ We show that KKPK employs an unusual binding mechanism that is entirely driven by entropic gains upon binding. We also compared our findings with the experimental data.

## 2 Methods

### 2.1 Theory

The main purpose of this study is to probe the energetics of binding of the minNLS KKPK peptide to impα. According to our previous simulations, KKPK binds diffusively to impα without forming well-defined binding poses. To estimate its binding free energy, Δ*G*_b_°, we used the double decoupling absolute FEP method^15^ combined with all-atom isobaric−isothermal replica exchange MD with solute tempering (REST).^20^

Because the FEP method has been extensively discussed in the literature,^15,18,32−34^ we include below only its summary. The standard binding free energy is related to the equilibrium constant, *K*_eq_
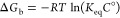
1where *R* is the gas constant, *T* is the temperature, and *C*° = 1/1661 Å^−3^ is the standard concentration. *K*_eq_ can be defined as
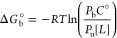
2where [*L*] is the concentration of the unbound ligands, *P*_b_ is the probability that one ligand is bound assuming that their number *N* ≫ 1, *P*_u_ is the probability that none of the ligands are bound, and *P*_u_ + *P*_b_ = 1. This ratio of probabilities can be written as a ratio of configurational integrals defining the reversible work of binding a ligand
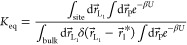
3where “site” and “bulk” represent bound and unbound states, *U* is the potential energy of the system, β = 1/*RT*,  are the coordinates of a bound ligand, and  are the remaining atoms’ coordinates. The delta function defines an arbitrary location for an unbound ligand  in the bulk. In our simulations, the minNLS peptide represents a ligand.

**Figure 1:**
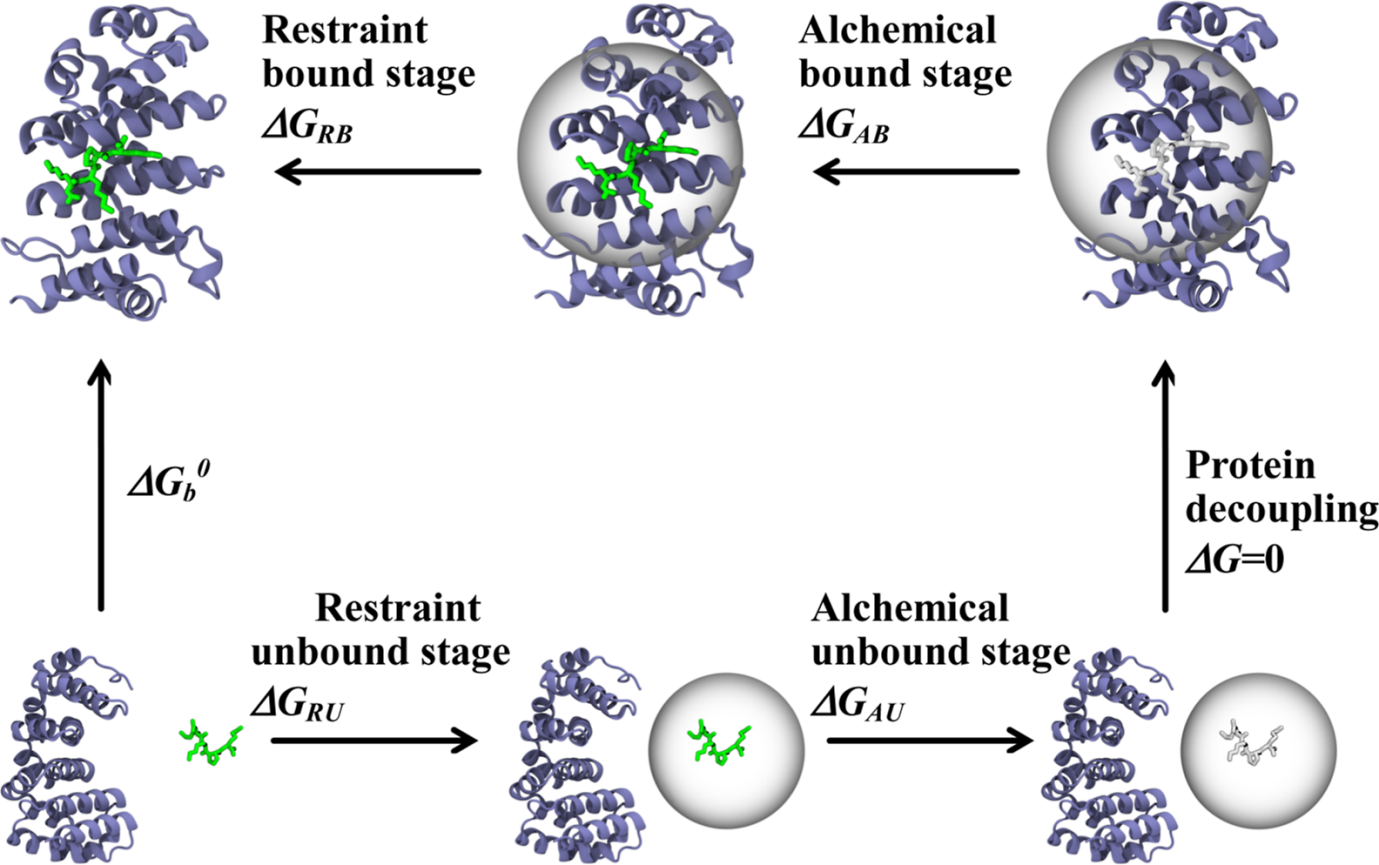
Thermodynamic cycle outlining the four stages used to estimate the free energy of binding Δ*G*_b_^°^. Impα protein is shown in ice blue cartoon representation, and the minNLS peptide is in green licorice when interacting with the environment or in white licorice when decoupled. The RB stage captures the release of the sphere restraint on the peptide, and AB and AU stages capture the free energy cost of coupling the peptide in the bound and its decoupling in the unbound states. The RU step captures the free energy cost of restraining the peptide in the unbound state. Decoupling of impα from the decoupled peptide does not change the free energy.

Following the FEP method, we constructed the thermodynamic cycle, shown in Figure 1, which decomposes the binding process into four stages. Consequently, eq 3 is partitioned into the evaluations over each of the cycle stages. Clockwise, the stages in the thermodynamic cycle are (i) releasing spherical restraint to a bound peptide (Restraint Bound, RB) Δ*G*_RB_, (ii) alchemical coupling of the bound peptide (Alchemical Bound, AB) Δ*G*_AB_, (iii) alchemical decoupling of the unbound peptide (Alchemical Unbound, AU) Δ*G*_AU_, and (iv) restraining the unbound peptide to the sphere Δ*G*_RU_ (Restraint Unbound, RU). Accordingly, eq 3 becomes
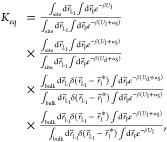
4where *U*_l_, *U*_d_, and *u*_S_ are the energies of the system with coupled and decoupled ligands, and of the restraining sphere, respectively. With the free energy changes defined for each stage, the equilibrium constant can be written as
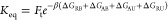
5where *F*_t_ is a factor representing the volume available to the ligand in the simulations. Then, the standard free energy of binding, Δ*G*_b_°, is
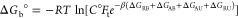
6

### 2.2 Restraint Design

**Figure 2:**
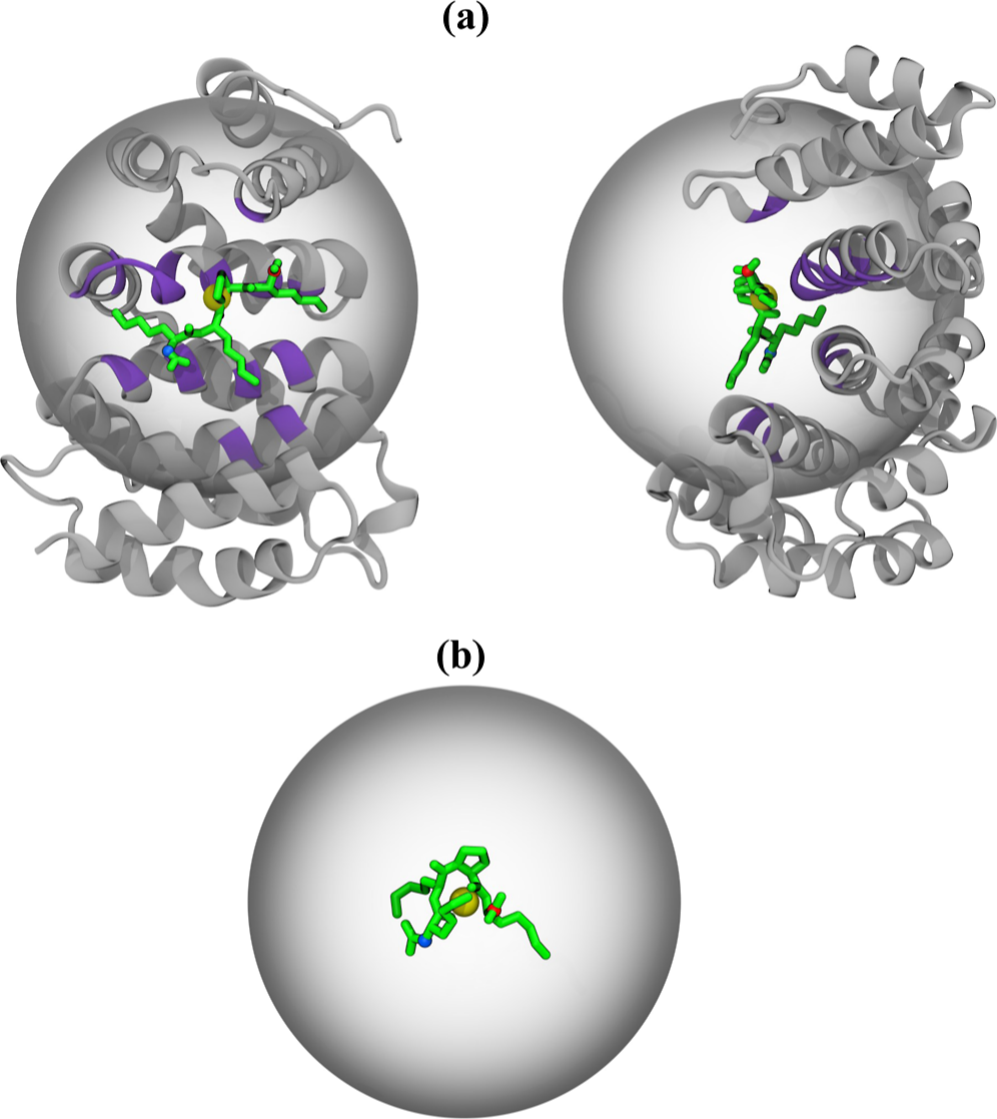
Two simulation systems, with KKPK peptide bound to impα (a) and unbound (b), are used along the thermodynamic cycle depicted in Figure 1. In (a), the spherical restraint in gray confines the minNLS peptide to the native minNLS binding site of impα shown in purple. This panel shows two views of the bound system. The same restraint is used for the unbound state in (b). The protein is shown in gray cartoon, and the minNLS peptide is in green licorice. The peptide termini are in blue and red. The restraining sphere center is represented by a yellow dot slightly offset from the native minNLS binding site of impα. The minNLS peptide binds diffusively adopting a manifold of non-native poses.

Traditional FEP simulations assume that a ligand adopts a well-defined binding pose. Confining a ligand to this pose is accomplished with Karplus restraints.^18^ However, our previous simulations have shown that upon interaction with impα minNLS peptide behaves differently by forming a manifold of diverse binding poses.^29^ To accommodate this binding behavior, we designed a soft-walled flat-bottom harmonic spherical restraint, which confines a peptide to the minNLS binding site of impα defined in Section 2.7 (Figure 2a). The restraint was defined by the potential , with the force constant *K* = 10.0 kcal/mol/Å^2^ and *r*_0_ = 18 Å acting on the center of mass of the peptide located at the distance *r* from the sphere center. The sphere center was fixed in space. Thus, this restraint remains inactive unless *r* > *r*_0,_ allowing for unhindered exploration of the binding site but preventing the peptide from leaving it. The Colvars module in NAMD was used to implement the spherical restraint.^35^ To ensure that the restraining sphere continuously encompasses the minNLS binding site of impα, we apply Karplus-like restraints to the protein (see Supporting Information and Figure S1). We verified in Supporting Information that, on average, these restraints position the impα minNLS binding site within the sphere confining the peptide. Since the restraints imposed on impα are active throughout the entire thermodynamic cycle, their contributions to the free energy of binding are canceled.

### 2.3 Simulation Systems

Following the cycle in Figure 1, we built two simulation systems: one for the bound state shown in Figure 2a and one for the unbound shown in Figure 2b. The bound system contained the minNLS peptide, which represents the P2−P5 positions in the NLS VEEV capsid sequence,^29^ and murine impα protein in its native state (PDB ID 3VE6). Impα was truncated at residue 211 to reduce the number of atoms while preserving the major NLS binding site, which entails the armadillo repeats 2−4.^27^ We verified that truncation and absence of the IBB domain did not appreciably change the impα structure (see Section 2.7 and Supporting Information). The unbound system contained a single minNLS peptide in water bulk. Impα and the peptide in both systems had neutral acetylated and amidated caps. The bound and unbound systems had 7715 and 9045 water molecules, 24 and 26 Na and 22 and 29 Cl ions to neutralize the net charge. The NaCl concentration was 150 mM in both systems. For both systems, the initial box size was set to 60 Å × 60 Å × 80 Å. After initial equilibration, the bound and unbound systems’ box sizes were 58.1 Å × 58.1 Å × 77.5 Å and 58.8 Å × 58.8 Å × 78.4 Å. All systems used CHARMM36m protein force field^36^ to parametrize the impα protein and the minNLS peptide. Water molecules were modeled with CHARMM-modified TIP3P.^37,38^

### 2.4 FEP/REST Simulations

To improve sampling and convergence in our simulations, we used the isobaric−isothermal replica exchange with solute tempering MD coupled to free energy perturbation (FEP/REST). Because REST formulation is detailed in the literature,^20^ we provide solely an overview of combining REST with FEP. FEP/REST involves *R* replicas simulated in parallel at conditions *m* characterized by temperature *T*_*m*_ and coupling parameter λ_*m*_ (0 ≤ *m* ≤ *R* − 1). In our version of the REST algorithm, we applied Hamiltonian scaling to the interactions between solvent atoms and between solvent and solute. We defined the solute as the peptide KKPK and the solvent as impα, water, and ions. As a result, although the replicas are simulated at the temperatures *T*_*m*_, Hamiltonian scaling sets the effective temperature of the solvent to *T*_0_. Thus, REST tempers solute, but keeps the solvent “cold”. REST scaling leads to significant reduction in the number of replicas without significant degradation of sampling.^39^ The FEP/REST enthalpy is
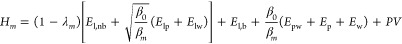
7where *E*_l,nb_ and *E*_l,b_ are the energies of the ligand nonbonded and bonded interactions, *E*_p_ is the protein’s energy, *E*_w_ is the energy of water, *E*_lp_, *E*_lw_, and *E*_pw_ are the energies of the ligand–protein, ligand–water, and protein−water interactions, *P* and *V* are the pressure and volume of the system, , and λ_*m*_ is the scaling parameter coupling the ligand with the environment. Following prior studies, we did not apply alchemical scaling to ligand bonded interactions.^40,41^ It should also be noted that NAMD does not apply REST scaling to Urey−Bradley and NBFIX terms in the energy function. When λ_*m*_ = 0, the ligand is fully coupled, whereas at λ_*m*_ = 1, the ligand is fully decoupled. The scaling factor  strengthens solvent interactions, effectively decreasing its temperature to *T*_0_. Exchanges between replicas *r* and *r* + 1 at the conditions *m* and *m* + 1 are attempted every 2 ps, and the probability of accepting a swap is given by the Metropolis criterion with the probability ω = min[1,*e*^−Δ^], where Δ is
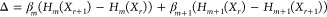
8and *X* represents system atom positions.

All simulations were completed using NAMD3.^42^ The integration step was set at 1 fs to ensure accuracy in the free energy computations. All hydrogen covalent bonds were constrained with ShakeH. Electrostatic interactions were handled with an Ewald summation. van der Waals interactions were smoothly truncated in the interval of 8 to 12 Å. Underdamped Langevin dynamics with the damping coefficient γ = 5 ps^−1^ was used for temperature control. The pressure was set to 1 atm using the Nosé–Hoover Langevin piston method with a piston period of 200 fs and a decay of 100 fs. All dimensions of the simulation systems were coupled.

#### 2.4.1 Alchemical and Restraint Simulation Stages

The RB simulations in the thermodynamic cycle release the spherical restraint on the bound peptide. In these FEP/REST simulations, λ_*m*_ scales the force constant *K* of the sphere restraint *u*_S_. Table S1 in Supporting Information lists the *R* = 15 unique conditions (λ_*m*_, *T*_*m*_), illustrating a steep reduction in the force constant, while the temperatures follow the usual geometric distribution peaking at λ_7_. We produced five 60 ns RB trajectories and used different sets of initial structures, all of which came from the equilibrated portion of previous REST binding simulations.^29^ The total RB sampling amounts to 4.5 μs, of which 2.1 μs is considered equilibrated (see Supporting Information). AB and AU simulations couple or decouple the peptide from the environment in the bound or unbound states, respectively. The corresponding alchemical transformations are determined by the conditions (λ_*m*_, *T*_*m*_) listed in Tables S2 and S3. AB and AU employed *R* = 40 and *R* = 32 conditions, respectively. In these simulations, λ_*m*_ varied from 0 to 1, while *T* peaked at λ_*m*_ = 0.5. Due to the number of atoms undergoing decoupling, the conditions near λ_*m*_ = 1 have smaller coupling increments to promote convergence. A soft-core van-der-Waals shifting coefficient of 5 Å^2^ was used to prevent overlap of alchemical atoms.^43^ We completed three 72 ns FEP/REST trajectories for AB and three 48 ns trajectories for AU. The total AB and AU samplings reached 8.64 and 4.608 μs, respectively. As shown in Supporting Information, equilibrium sampling in AB and AU simulations amounts to 3.84 and 4.608 μs, respectively. Similarly to the RB simulations, we used the structures from our previous work to initialize AB.^44^ For AU trajectories, we performed a preliminary 10 ns equilibration *NPT* simulation followed by a 25 ns 700 K *NPT* simulation intended to randomize the peptide in water. To compute changes in free energy or enthalpy along the thermodynamic cycle, we used the multistate Bennett acceptance ratio methodology with pymbar 4.0.2.^45^ Details about technical performance and convergence of FEP/REST simulations can be found in the Supporting Information (Figures S2–S6).

### 2.5 Restraint Unbound Stage

The RU stage of the thermodynamic cycle estimates the free energy cost Δ*G*_RU_ of restraining the peptide in the unbound state. It was evaluated numerically by assuming that the peptide is subject to the restraining potential *u*_S_
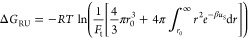
9

Integration was performed using a trapezoidal rule using the segment size of 0.05 Å. Note that the *F*_t_ factor cancels out from Δ*G*_b_^0^.

### 2.6 Corrections for Finite-Size and Discrete Solvent Effects

During alchemical decoupling of the charged peptide, there is a net charge change in the system. This net charge difference creates electrostatic artifacts due to periodic boundary conditions. The majority of these artifacts are driven by the finite size of the simulation system. Several methods have been proposed to address these artifacts. One of them is based on including coalchemical ions, which preserve the neutral charge of the system. However, KKPK ligand requires three alchemical counterions, which may bind to the peptide or impα in the course of FEP/REST simulations complicating convergence. Fortunately, there exists an alternative electrostatic correction to binding free energy alleviating these artifacts, which is as accurate as coalchemical ion approach.^46−48^ Following Rocklin et al.,^46^ we considered five contributions to ΔΔ*G*_b_, namely
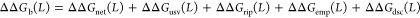
10where ΔΔ*G*_net_ corrects for periodicity induced net charge interactions, ΔΔ*G*_usv_ is a periodicity induced net charge undersolvation correction, ΔΔ*G*_rip_ corrects for residual integrated potential effects, ΔΔ*G*_emp_ is an empirical correction to ΔΔ*G*_rip_, ΔΔ*G*_dsc_ is a discrete solvent correction caused by application of implicit solvent, and *L* is a system dimension. The terms in eq 10 are computed as a difference between the corrections in the bound and unbound states. Computations of ΔΔ*G*_rip_ and ΔΔ*G*_emp_ utilize Poisson–Boltzmann equation and were accomplished with the APBS software.^49^ Following the parameters described by Rocklin et al., we used *L*_ref_ = 120 Å, a probe-contact surface *s*_rad_ = 1.4 Å, the interior permittivity *p*_die_ = 1.0, and the exterior permittivity for TIP3P water *s*_die_ = ϵ_S_ = 97.0. A sample APBS input file containing the complete set of parameters is provided in GitHub. Due to the variable box size in our *NPT* simulations, we used the average volume in each trajectory. APBS computations involve the sample of 24,000 equilibrated structures for each of the two states, bound and unbound.

### 2.7 Structural Probes

To study minNLS conformational ensemble, we followed our past publications^29^ and computed several structural probes using VMD.^50^ We defined that a contact between amino acids occurs if at least one pair of their heavy atoms is less than 4.5 Å apart. The native binding site of minNLS to impα was determined using the PDB 3VE6 structure and our contact definition. Then, the native minNLS binding site is composed of residues Ser35, Phe68, Trp72, Thr75, Asn76, Ala78, Ser79, Gly80, Thr81, Ser82, Thr85, Gln111, Trp114, Asn118, Asp122, Asn158, and Trp161. The minNLS peptide is assumed to be bound to impα if it forms at least one contact with the protein. A binding contact between minNLS and impα amino acids is native if it is present in the 3VE6 structure. Otherwise, the binding contact is non-native. Using these definitions, we computed the fraction of native contacts *P*_n_(*j*) formed by minNLS residues *j*. If *P*_n_(*j*) = 1.0, then *j* maintains all of its native binding interactions during the simulation. Then, *P*_nn_(*j*) is the fraction of non-native contacts out of all formed by *j*. If *P*_nn_(*j*) = 1.0, residue *j* forms only non-native binding interactions. The contact vector *P*_c_(*i*) collects the probabilities for impα residues *i* to establish binding interactions. To compute the number of water molecules in the first solvation shells (FSS), we analyzed radial distributions of water around lysine nitrogen atoms and determined that FSS extends up to *r*_s_ = 3.75 Å. The numbers of water molecules solvating charged side chains were computed considering the FSS of respective oxygen or nitrogen atoms. Secondary structure was computed using STRIDE.^51^Figure S7 in the Supporting Information demonstrates that the impα structure remains native-like during the simulations despite truncation and absence of IBB domain. The structural probes are reported as averages at *T*_0_ = 310 K. The sampling errors are computed as the standard error of the mean, taking each trajectory as an independent sample.

### 2.8 Conformational Clustering

We clustered the bound peptide structures using the density-based conformational clustering method from Daura et al.^52^ Our procedure was as follows: (1) align pairs of impα structures using the minimal RMSD computed between the heavy atoms of the minNLS binding site side chains, (2) after protein alignment, compute the RMSD between the peptide poses, and (3) apply Daura et al.’s clustering method to find clusters and their populations. We sampled 16,000 structures periodically from equilibrated simulations. We opted to use a cutoff of *R*_c_ = 2 Å. For analysis, we retained clusters with the populations of at least 1%, which together make up at least 50% of the structures in the data set.

## 3 Results and Discussion

### 3.1 Probing KKPK Binding Energetics Using FEP/REST

Using the thermodynamic cycle shown in Figure 1, FEP/REST simulations, and numerical integration, we computed the changes in the free energy of the system occurring upon releasing spherical restraint to a bound peptide (Restraint Bound, RB) Δ*G*_RB_, alchemical coupling of the bound peptide (Alchemical Bound, AB) Δ*G*_AB_, alchemical decoupling of the unbound peptide (Alchemical Unbound, AU) Δ*G*_AU_, and restraining the unbound peptide to the sphere Δ*G*_RU_. The respective free energy changes are summarized in Table 1. It follows that the release or introduction of the restraint makes minor contributions compared to the alchemical stages. Indeed, Δ*G*_RB_ is negative reflecting the costs of confining the peptide to the sphere, but its small value implies that the bound KKPK rarely attempts to escape the minNLS binding site. The negative and larger Δ*G*_RU_ indicates that the effective concentration of the peptide in the confining sphere is actually lower than in the standard state, resulting in entropic gain. Markedly dominant contributions to Δ*G*_b_^°^ come from alchemical transformations. Coupling the peptide to the bound state results in highly negative Δ*G*_AB_, which is attributed to favorable interactions formed by KKPK in the bound state compared to the vacuum. However, Δ*G*_AB_ is almost offset by the decoupling of KKPK in water. The net result of these thermodynamic transitions is the free energy of binding Δ*G*_b_^°^ = −9.3 kcal/mol listed in Table 2.

**Table 1:** Free Energy Changes along the Thermodynamic Cycle

stage	value, kcal/mol
Δ*G*_RB_	−0.06 ± 0.04
Δ*G*_AB_^a^	−155.23 ± 0.08
Δ*G*_AU_^a^	147.70 ± 0.08
Δ*G*_RU_^b^	−1.69

a The terms computed without Rocklin et al. corrections.

b Computed using numerical integration.

**Table 2:** Energetics of KKPK Binding to impα

term	before correction^a^^,^^b^, kcal/mol	after correction^b^^,^^c^, kcal/mol
Δ*G*_b_°	−9.3 ± 0.1	−4.8 ± 0.1
Δ*H*	−4.3 ± 1.8	0.1 ± 1.8
Δ*E*_vdw_	−5.4 ± 0.9	−5.4 ± 0.9
Δ*E*_el_	2.4 ± 1.6	6.8 ± 1.6
Δ*E*_bond_	−1.2 ± 1.5	−1.2 ± 1.5
*T*Δ*S*	4.9 ± 1.9	4.9 ± 1.9

a The terms computed without Rocklin et al. corrections.

b Due to rounding, the terms may not exactly add up.

c The terms adjusted by Rocklin et al. corrections.

Because our simulations are performed by using periodic boundary conditions and involve alchemical decoupling or coupling of a charged ligand, Δ*G*_b_^°^ must be corrected for finite-size and discrete solvent effects. Following the analytical correction scheme proposed by Rocklin et al.,^46^ we computed the correction to free energy ΔΔ*G*_b_, which includes periodicity induced net charge interactions term ΔΔ*G*_net_, periodicity induced net charge undersolvation term ΔΔ*G*_usv_, residual integrated potential contribution ΔΔ*G*_rip_, empirical term ΔΔ*G*_emp_ correction to ΔΔ*G*_rip_, and the correction for discrete solvent effects ΔΔ*G*_dsc_. These corrections are given in Table 3 and reveal that two of them, ΔΔ*G*_rip_ and, particularly, ΔΔ*G*_dsc_, significantly impact the binding free energy. Indeed, due to the large positive charge of KKPK and relatively small dimensions of the unit cell, ΔΔ*G*_rip_ = −1.4 kcal/mol, whereas driven by a sizable solvent-excluded volume, ΔΔ*G*_dsc_ is 5.9 kcal/mol. As a result after correction, the free energy of binding Δ*G*_b_^°^ is reduced to −4.8 kcal/mol (Table 2). This outcome indicates that the KKPK peptide has a moderate binding affinity to impα.

**Table 3:** Corrections to Binding Energetics due to Finite-Size and Discrete Solvent Effects^a^

term	value, kcal/mol
ΔΔ*G*_b_	4.4 ± 0.0
ΔΔ*G*_net_	0.9 ± 0.0
ΔΔ*G*_usv_	−0.9 ± 0.0
ΔΔ*G*_rip_	−1.4 ± 0.0
ΔΔ*G*_emp_	0.0 ± 0.0
ΔΔ*G*_dsc_	5.9 ± 0.0

a Corrections are computed according to Rocklin et al.^46^

To understand the binding mechanism, we evaluated different contributions to the Δ*G*_b_^°^. It follows from Table 2 that the enthalpy Δ*H* makes a minor (within the error) contribution to binding. Interestingly, its further decomposition indicates that the gain in van der Waals interactions Δ*E*_vdw_ is highly favorable for binding, but it is more than offset by the unfavorable change in electrostatic energy Δ*E*_el_. There is also a minor favorable contribution to peptide binding from bonded interactions, suggesting that the KKPK bound conformation is more relaxed than the unbound. These findings imply that a highly charged KKPK and, possibly, charged impα amino acids make strong attractive electrostatic interactions with water, which are partially lost in the bound state. Although the peptide−protein complex gains attractive van der Waals binding interactions, they do not compensate for the loss in hydration. Strikingly, Table 2 demonstrates that the sole factor ultimately driving KKPK binding is the favorable entropic change *T*Δ*S*. Taking also into account a large loss in electrostatic interactions, we surmise that KKPK binding must result in a significant release of water molecules from the peptide and impα solvation shells, increasing the overall entropy.

**Figure 3:**
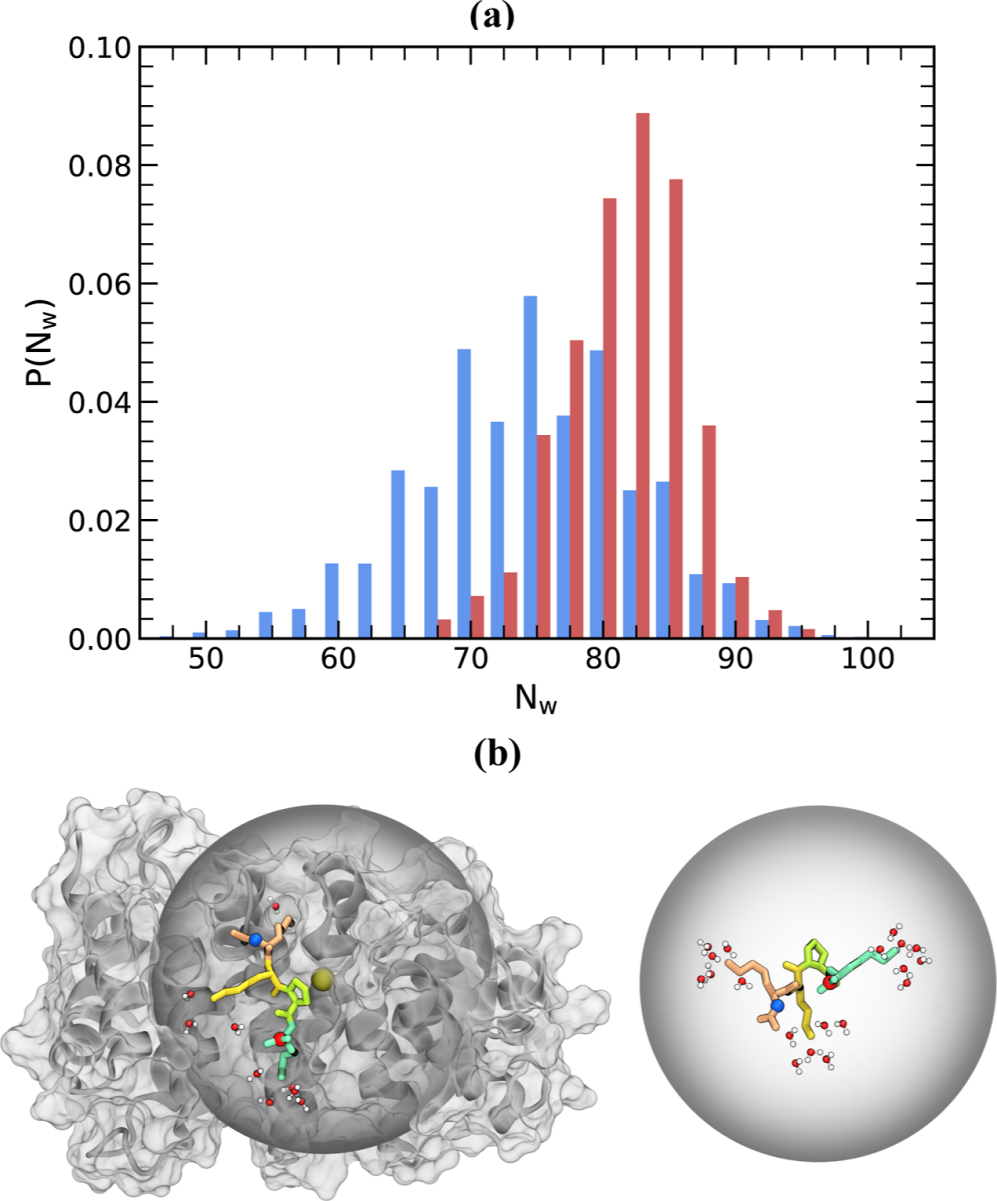
(a) Probability distributions *P*(*N*_w_) of the numbers of water molecules in the FSS of charged amino acids in the bound (in blue) and unbound (in red) states. (b) Water molecules in the FSS of minNLS lysine amino acids in the bound and unbound states are shown. In both panels, impα minNLS binding site with the bound minNLS peptide represents the bound state, whereas the unbound state is given by unbound peptide and the empty impα minNLS binding site. The minNLS peptide is in licorice representation, whereas impα including its backbone and solvent accessible surface is in gray. The figure demonstrates the loss of water in the solvation shells of charged side chains, particularly of Lys6, upon peptide binding to impα.

To investigate the origin of entropic gains, we probed the release of water upon binding of KKPK to impα (see Methods and Supporting Information). The sampling of unbound peptide comes from AU simulations at condition *m* = 0 (λ_0_ = 0, *T*_0_ = 310 K), whereas condition *m* = 39 (λ_39_ = 1, *T*_39_ = 310 K) in AB simulations provides sampling of unbound impα. The loss of electrostatic interactions Δ*E*_el_ suggests the desolvation of charged amino acids upon binding. Consequently, from these simulations, we computed the number of water molecules residing in the FSS of minNLS and impα charged residues in the unbound state, *N*_w_(*u*). We also computed the number of water molecules in the FSS of charged residues for the bound complex, *N*_w_(*b*). To this end, we used condition *m* = 0 (λ_0_ = 0, *T*_0_ = 310 K) in AB simulations. The corresponding probability distributions *P*(*N*_w_) of the numbers of FSS water molecules are shown in Figure 3a. In the unbound state, the average number of water molecules in the FSS of charged residues is ⟨*N*_w_(*u*)⟩ = 82.7, but is reduced to 75.4 when the peptide is bound. Thus, binding of KKPK to impα releases ⟨Δ*N*_w_⟩ = 7.3 water molecules, as illustrated in Figure 3b. If we restrict these computations to the FSS of minNLS lysines, then ⟨Δ*N*_w_⟩ is reduced to 3.0. It is easy to show that when a single water molecule is transferred from the Lys FSS in the unbound state to a standard state, the gain in entropy amounts to 1.2 kcal/mol. This simple computation shows that a release of water molecules solvating Lys may be almost sufficient to explain the entropy gain upon KKPK binding. It should be noted that the actual contribution of water to *T*Δ*S* upon binding is partially offset by the temporal confinement of water to FSS as well as by changes in translational, rotational, and vibrational entropies of KKPK and impα.

It is noteworthy that KKPK binding results in the release of a mere 10% of all water molecules present in the FSS of charged amino acids. Thus, peptide binding does not shield them completely from water. However, different amino acids exhibit dramatically different desolvation. From Figure S9 in Supporting Information, we obtain ⟨Δ*N*_w_⟩ for individual lysine amino acids in KKPK. Lys6 ⟨Δ*N*_w_⟩ = 2.4, implicating a 50% loss of FSS water. Lys9 loses about 1 water molecule or 20% of FSS water, whereas Lys7 ⟨Δ*N*_w_⟩ = 0.4 or 7% loss. Therefore, the largest contribution to the loss of FSS water from lysine amino acids comes from Lys6 (up to 80%), followed by Lys9, while the contribution of Lys7 is small. If binding of KKPK to impα is driven by entropic gains, then mutation of Lys6 should considerably reduce the minNLS binding affinity due to a reduced gain in water entropy. It is known that Lys6 at the position P2 in the conserved NLS motif K-K/R-X-K/R makes the largest contribution to binding energetics due to formation of a salt bridge with impα Asp122.^27^ Our simulations suggest a complementary reason for the importance of this NLS amino acid for binding.

An additional factor favoring binding entropy gain should be noted. According to our previous simulations^29^ and the analysis of FEP/REST binding ensemble presented below, the KKPK peptide does not form a single binding pose instead adopting a manifold of non-native poses. Hence, the two factors outlined in this section support favorable changes in binding entropy, and we conclude that the binding mechanism of KKPK peptide is purely entropic.

### 3.2 Analysis of Binding Structural Ensemble

As a rule, FEP simulations are used solely to obtain binding energetics. However, its reliable evaluation requires extensive sampling of ligand bound structures. Consequently, it should be possible to extract from FEP/REST simulations not only binding energetics but also conformational properties of the minNLS peptide. In this section, we test this possibility by comparing the binding ensembles produced by our FEP/REST simulations and earlier exhaustive REST sampling.^29^ To this end, we combined the equilibrium sampling collected in AB and RB simulations under the condition *m* = 0 (λ_0_ = 0, *T*_0_ = 310 K). Collectively, these simulations produced 236 ns of *m* = 0 sampling. Previous REST simulations probed minNLS binding to the major NLS binding site at 310 K for 240 ns and serve as reference.^29^

First, we compare the general properties of bound KKPK sampled in both simulations. Table 4 shows that in FEP/REST simulations KKPK amino acids *j* retain, on average, about 30% of native binding interactions observed in the 3VE6 PDB structure. Their largest fraction, *P*_n_(*j*), is observed in the N-terminus, but it largely vanishes at the C-terminus. Driven by a non-native binding of the C-terminus, close to a half of binding interactions formed by KKPK are non-native. The distribution of binding interactions along the minNLS sequence ⟨*C*(*j*)⟩ shows that *j* = Lys6 has the largest binding affinity, followed by *j* = Lys9 and, with a large gap, by *j* = Lys7 and Pro8, whose ⟨*C*(*j*)⟩ are about threefold weaker than of *j* = Lys6. The comparison of the FEP/REST and REST simulations in Table 4 leads to the following conclusions. In FEP/REST and REST simulations, the fractions of retained native interactions *P*_n_(*j*) and their average are identical within the sampling errors, and both suggest that KKPK does not adopt a native pose upon binding to impα. The average fractions of non-native interactions *P*_nn_ collected in FEP/REST and REST also match within error, while indicating a non-native binding of the minNLS in the C-terminus. Finally, the distributions of binding interactions ⟨*C*(*j*)⟩ along the minNLS reveal overlapping error intervals for all but one *j*.

**Table 4:** Binding Interactions Formed by the minNLS Peptide

	Lys6	Lys7	Pro8	Lys9	Peptide
FEP/REST sampling					
*P*_n_(*j*)^a^	0.53 ± 0.07	0.27 ± 0.04	0.24 ± 0.04	0.12 ± 0.03	0.33 ± 0.04
*P*_nn_(*j*)^b^	0.29 ± 0.05	0.22 ± 0.04	0.48 ± 0.07	0.71 ± 0.04	0.47 ± 0.05
⟨*C*(*j*)⟩^c^	6.1 ± 0.7	2.2 ± 0.3	2.3 ± 0.2	3.7 ± 0.2	14.3 ± 0.4
REST sampling^d^					
*P*_n_(*j*)^a^	0.51 ± 0.07	0.24 ± 0.04	0.22 ± 0.04	0.11 ± 0.02	0.31 ± 0.04
*P*_nn_(*j*)^b^	0.40 ± 0.03	0.27 ± 0.09	0.65 ± 0.12	0.86 ± 0.04	0.54 ± 0.07
⟨*C*(*j*)⟩^c^	7.6 ± 0.8	1.9 ± 0.2	1.8 ± 0.4	4.6 ± 0.5	16.0 ± 0.7

a Average fraction of native contacts, i.e., those formed by amino acid *j* in the 3VE6 structure and observed in the FEP/REST simulations.

b Average fraction of non-native contacts formed by amino acid *j*.

c Average number of binding contacts formed by amino acid *j* with impα.

d Data reported previously.^29^

Second, we analyzed the locations of KKPK binding on the impα surface. To this end, we computed the list of 10 impα amino acids *i* with the largest probabilities of interaction with KKPK, *P*_c_(*i*). Table 5 compares the *P*_c_(*i*) computed using FEP/REST and REST sampling. It follows from the table that apart from one amino acid, both simulations identify the same 10 impα amino acids with the highest *P*_c_(*i*). Moreover, the top three amino acids are the same, and for seven amino acids, *P*_c_(*i*) matches within their sampling errors. In both simulations, 8 out of 10 amino acids belong to the native minNLS binding site including two tryptophan residues, Trp114 and Trp161, and anionic Asp122. The inclusion of these amino acids is important, because tryptophan residues form a cage for minNLS Lys side chain, whereas Asp122 establishes a strong electrostatic contact with minNLS Lys6.^27^ Taken together, Tables 4 and 5 demonstrate that in both simulations, KKPK peptide largely maintains binding to the impα major NLS binding site but loses most of its native binding interactions. This result is consistent with the diffusive binding of the peptide.

**Table 5:** Top 10 Binding impα Amino Acids^a^^,^^b^

rank	FEP/REST	REST^c^
1	**Trp161** (0.91 ± 0.01)	**Trp161** (0.96 ± 0.00)
2	**Asn118** (0.80 ± 0.04)	**Ser79** (0.83 ± 0.01)
3	**Ser79** (0.80 ± 0.04)	**Asn118** (0.77 ± 0.03)
4	**Gly80** (0.69 ± 0.08)	Asp200 (0.73 ± 0.01)
5	**Ala78** (0.67 ± 0.08)	**Gly80** (0.69 ± 0.03)
6	**Asp122** (0.67 ± 0.07)	**Ala78** (0.68 ± 0.03)
7	**Thr85** (0.66 ± 0.08)	**Thr85** (0.68 ± 0.03)
8	**Trp114** (0.65 ± 0.07)	**Asp122** (0.67 ± 0.04)
9	Gly121 (0.56 ± 0.08)	**Trp114** (0.66 ± 0.02)
10	Asp200 (0.48 ± 0.08)	Glu196 (0.60 ± 0.01)

a Amino acids belonging to the native minNLS binding site are in bold.

b Probabilities *P*_c_(*i*) for impα amino acid *i* to bind KKPK are in parentheses.

c Data reported previously.^29^

**Figure 4:**
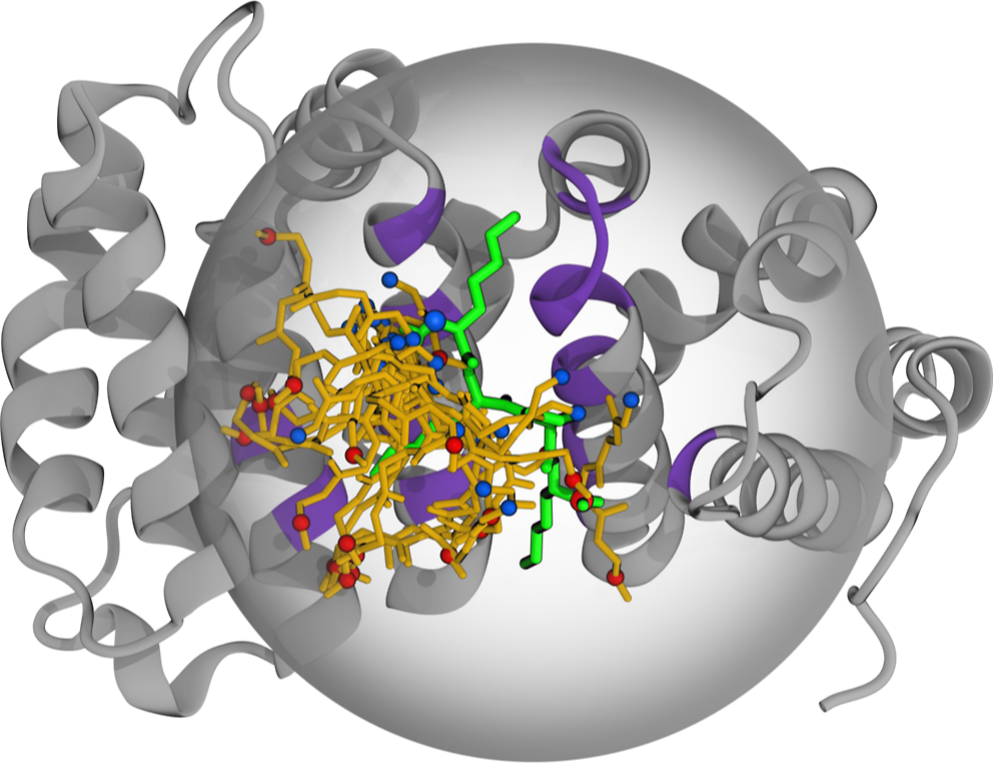
Distribution of KKPK backbone binding poses. Impα is shown in gray with the minNLS peptide in native bound pose shown in green. The protein and the peptide are extracted from 3VE6 structure. The sphere restraining the minNLS encloses the native minNLS binding site shown in purple. MinNLS bound poses in yellow represented by the centroids of 24 bound clusters sampled by FEP/REST are superimposed on the native pose. The N- and C-termini of KKPK are in blue and red. The figure illustrates highly diffusive binding of the minNLS peptide to impα.

**Figure 5:**
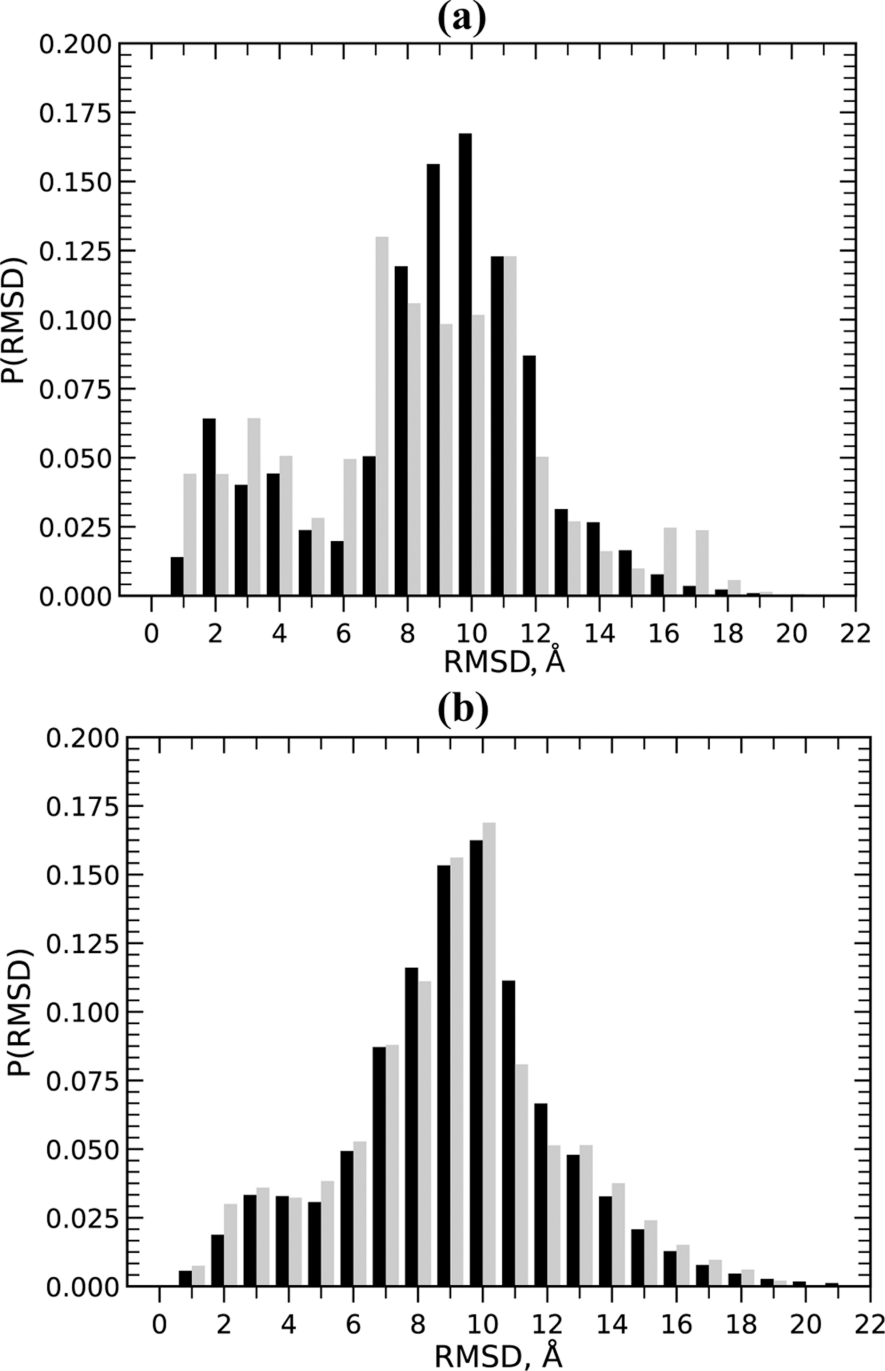
(a) Probability distributions of peptide RMSD values *P*(RMSD) computed against its native pose in 3VE6 structure. (b) Probability distributions *P*(RMSD) of RMSD values comparing bound minNLS structures all-vs-all. In both panels, data in black and gray represent FEP/REST and REST^29^ simulations. The figure shows a good agreement between both simulations in sampling highly heterogeneous non-native binding ensembles for the minNLS peptide.

If the minNLS retains few native binding interactions but its overall probability of binding impα *P*_b_ ∼ 1.0, does this peptide adopt a single non-native binding pose or a multitude of different binding poses? To answer this question, we clustered KKPK conformations bound to impα, as described in Methods. We analyzed the top populated clusters, which together capture more than 50% of structures in the minNLS binding ensemble. Table S4 lists 24 clusters observed in the FEP/REST simulations. Their distribution shows a highly heterogeneous bound ensemble, in which none of the clusters has a population *P*_cl_ exceeding 0.1. The cluster ranked #1 is native with the RMSD from the native pose of 2.0 Å, but it barely collects the fraction of 0.08 of binding poses. Of 23 remaining clusters 19 have the RMSD from the native pose exceeding 7.0 Å. Figure 4 illustrates the highly diffusive binding of the minNLS peptide to impα. A non-native binding of the minNLS is confirmed by the probability distribution of peptide RMSD values *P*(RMSD) computed against its native pose in the 3VE6 structure. Indeed, Figure 5a shows that *P*(RMSD) computed using FEP/REST simulations is broad with the average RMSD of 8.8 Å. To directly map the heterogeneity in the bound peptide ensemble, we obtained the probability distribution *P*(RMSD) of RMSD values computed between all minNLS bound structures. In Figure 5b, this all-vs-all distribution is broad with an average of 9.2 Å, indicating highly diverse bound conformations. Thus, FEP/REST simulations implicate a highly non-native, heterogeneous binding ensemble for KKPK peptide consistent with diffusive binding.

For comparing FEP/REST and REST simulations, we first considered the clusters in the bound ensembles. Table S5 presents an all-vs-all structural comparison of the FEP/REST and REST clusters. If we select the cutoff RMSD of 3.0 Å to assume a moderate structural similarity between cluster centroids, then 16 out of 24 FEP/REST clusters (or 67%) match REST clusters. Likewise, 11 out of 14 REST clusters (or 79%) find their counterparts in the FEP/REST ensemble. We next compared the probability distributions of peptide RMSD values *P*(RMSD) computed against the native pose in the 3VE6 structure. Figure 5a shows that although FEP/REST and REST *P*(RMSD) do not match perfectly, both exhibit the highest probabilities between approximately 7 and 12 Å. The average RMSD from the 3VE6 structure of 8.8 Å in FEP/REST and 8.3 Å in REST simulations are also close. The FEP/REST and REST probability distributions *P*(RMSD) comparing all-vs-all peptide bound structures in Figure 5b are remarkably similar. Indeed, the average RMSD values for FEP/REST and REST simulations are 9.2 and 9.0 Å, respectively. Therefore, despite some variances, both FEP/REST and REST simulations collected consistent, highly heterogeneous bound ensembles featuring mostly non-native peptide poses.

### 3.3 Broader Outlook

The broader implications of our work are twofold. First, we have designed an absolute FEP protocol that includes REST enhanced sampling and spherical harmonic restraint. In contrast to traditional FEP restraints, which confine a ligand to a specific pose, our restraint imposes no restrictions on the orientations, positions, or conformations of a ligand within the sphere. As a result, the newly developed protocol handles well the ligands binding diffusively without forming specific binding poses. Our previous research has shown that the minNLS peptide KKPK belongs precisely to this class of ligands.^29^ The estimate of its binding free energy, Δ*G*_b_^°^, which has converged in the course of FEP/REST simulations, and the analysis of binding energetics demonstrated the utility of our protocol. Additionally, our extensive comparisons of the binding ensembles sampled by REST and FEP/REST simulations reveal their good agreement. As with any complex all-atom simulations there are sampling variations between FEP/REST and REST binding ensembles, but both point at the same properties of minNLS binding to impα—(i) a broad distribution of diverse binding poses within the limits of the major NLS binding site in impα and (ii) a dominance of non-native binding interactions with small contribution from native contacts. An alternative to FEP/REST approach for computing Δ*G*_b_^°^ based on constructing the potential of mean force profile along a binding reaction coordinate using umbrella-like simulations^15^ may also be extended to diffusively binding ligands by applying our spherical restraints. More generally, given that the exact amounts of entropic and enthalpic contributions to binding are rarely known a priori, our approach may be adopted as a safeguard against unintended errors in FEP calculations. A predominantly enthalpic binder is expected to exhibit a single binding pose similar to that observed for the coreNLS peptide KKPKKE.^29^ The estimations of Δ*G*_b_^°^ in these cases could be handled by traditional FEP approaches. Yet, when multiple binding poses or their continuous distribution determine Δ*G*_b_^°^, our FEP/REST protocol should provide a more accurate evaluation of binding affinity.

Second, using FEP/REST simulations, we studied the mechanism of KKPK binding to impα. We found that the binding mechanism for this peptide is purely entropic driven by an increase in system entropy and almost vanishing enthalpic change. The latter is associated with the loss of favorable electrostatic interactions between minNLS and impα charged side chains and water in the bound state, which is nearly compensated by the gain of van der Waals interactions. We traced the increase in entropy upon binding to partial release of water from the solvation shells of the charged peptide and impα amino acids. It is reasonable to expect that such a mechanism would be generally applicable to the binding of other cationic peptides to proteins. Indeed, prior experimental and computational studies support this assumption. For instance, peptide binding protein OppA is a part of oligopeptide permease system responsible for transporting peptide cargo into cell. Using isothermal titration calorimetry (ITC), Sleigh et al. measured the binding affinities of KXK peptides to OppA binding site, where X stands for any of 20 naturally occurring amino acids.^53^ Intriguingly, for all peptides, the enthalpy change upon binding was unfavorable (namely, for all Δ*H* > 0), whereas the corresponding entropic change favored binding (*T*Δ*S* > 0). Therefore, all these peptides apparently bind OppA via an entropic mechanism. Strikingly, the largest entropic gains *T*Δ*S* ≈ 17 kcal/mol have been seen for cationic KKK or KRK, whereas for KGK, *T*Δ*S* ≈ 11 kcal/mol is the third lowest value among 20 amino acids. Furthermore, KGK binding results in the enthalpic loss Δ*H* of about 3 kcal/mol, which is increased threefold to ≈9 kcal/mol for KKK. Both observations are consistent with the loss of water from the peptide (and protein) solvation shells. Although many factors influence these estimates, including the composition and topology of the OppA binding site, the findings of Sleigh et al. support our interpretation of the KKPK binding mechanism as driven by the release of water hydrating charged amino acids upon peptide binding.

To the best of our knowledge, prior FEP studies have not investigated enthalpic and entropic contributions to the binding free energy for charged ligands. The closest computational work is of Gilson and co-workers, who have probed the binding of nine ligands with different net charges to cucurbit[7]uril and β-cyclodextrin.^54^ The authors compared the binding free energies of the ligands as well as corresponding changes in enthalpy and entropy computed from the attach-pull-release free energy framework or extracted from ITC experiments.^55^ They found that a large net ligand charge generally results in a large positive *T*Δ*S*. For example, the ligand B11 with the net charge of +4 exhibits a favorable binding entropic change *T*Δ*S* = 12.7 kcal/mol, whereas the ligands A1 and B2 with zero net charges reveal entropic losses, i.e., their *T*Δ*S* < 0. The fact that the B11 binding energetics also included a large favorable enthalpic contribution not observed in our simulations indicates that the balance between entropic and enthalpic terms depends on subtle details of binding interfaces.

If the minNLS peptide binds diffusively to impα without forming a well-defined pose and its binding is driven by entropic gains from desolvation, then one may speculate that the minNLS sequence plays a minor role as long as it contains three lysine (or arginine) residues. Therefore, we expect that any peptide composed on three cationic and arbitrary amino acid X would bind to impα via the same mechanism as KKPK. The example could be KKRK fragment from the SV40 virus NLS sequence.^27^ Consistent with this hypothesis, KXK peptides, where X is any of 20 amino acids, strongly bind OppA with the free energies being in the narrow interval from −7.1 to −10.1 kcal/mol.^53^ It is likely that the entropic binding mechanism is active for highly charged peptides in general as long as their binding is governed by the loss of favorable electrostatic interactions with water and its release from the solvation shells of charged residues. It is also tempting to speculate on the utility of entropic binding mechanism for NLS recognition by impα. VEEV minNLS represents a conserved NLS fragment, which binds via an entropic mechanism. The “advantage” of such binding mechanism is that it is diffusive and nonspecific accommodating multiple binding poses. Yet, extending minNLS KKPK to the coreNLS KKPKKE makes its binding specific and native.^29^ One may suggest that NLS-based recognition of protein cargo employs the minNLS motif for initial docking of NLS to impα, because it does not require any specific pose but yet offers a moderate binding affinity. Only after minNLS docking has been accomplished, the NLS is locked into the native pose by recruiting the interactions of adjacent amino acids. This rationale may explain why the minNLS motif, which binds entropically, is conserved across nucleus-bound proteins.

## 4 Conclusions

In this paper, we have developed and tested an absolute FEP protocol FEP/REST, which combines all-atom MD, replica exchange with solute tempering (REST) enhanced sampling, and spherical harmonic restraint applied to a ligand. Our objective was to compute the binding free energy together with underlying binding mechanism for a ligand, which binds diffusively to a protein. Such ligands represent a nearly impossible target for traditional FEP simulations. As a case study, we selected a conserved motif KKPK termed minNLS from the NLS sequence of VEEV capsid protein. This peptide fragment binds diffusively to importin-α transport protein without forming well-defined poses. As a reference for conformational sampling, we used our prior exhaustive REST simulations probing the conformational ensemble of minNLS binding to importin-α. We showed that FEP/REST simulations with spherical restraint provide a converged estimate of the binding free energy. It shows that minNLS binds with moderate affinity to importin-α utilizing a purely entropic mechanism, in which binding free energy is determined by favorable entropic gain. For the cationic minNLS peptide, a favorable entropic gain upon its binding to importin-α is primarily associated with the release of water from the solvation shells of charged amino acids. We demonstrated that FEP/REST simulations sample the KKPK bound ensemble well, allowing us to characterize the distribution of bound structures, binding interactions, and locations on the importin-α surface. Analysis of experimental studies offers support to our rationale behind KKPK entropic binding mechanism.

## Data Availability

NAMD is available at https://www.ks.uiuc.edu/Research/namd/. VMD is available at https://www.ks.uiuc.edu/Research/vmd/. Initial structures, topology files, NAMD and APBS configuration files, and codes used for data analysis are available at https://github.com/KlimovLab/AFEP-REST_KKPK_impa.

## Supporting Information Available

Additional details concerning the model, methods, and results (PDF)
