# Supplementary material for: Applying Absolute Free Energy Perturbation Molecular Dynamics to Diffusively Binding Ligands
Source: J Chem Theory Comput. 2025 Apr 7;21(8):4286–98. doi: 10.1021/acs.jctc.5c00121 (PMC12020361; doi:10.1021/acs.jctc.5c00121)
Supplement: Supplementary file 1 — ct5c00121_si_001.pdf [file ct5c00121_si_001.pdf]

Supporting Information

Applying absolute free energy perturbation molecular dynamics to diffusively binding ligands

Xavier E. Laracuente[1], Bryan M. Delfing[1], Xingyu Luo[1], Audrey Olson[1], William Jeffries[1], Steven R. Bowers[1], Kenneth W. Foreman[2], Kyung Hyeon Lee[2,3], Mikell Paige[2,3], Kylene Kehn-Hall[4,5], Christopher Lockhart[1], and Dmitri K. Klimov[1*]

[1]School of Systems Biology, George Mason University, Manassas, VA 20110, USA
[2]Department of Chemistry and Biochemistry, George Mason University, Fairfax, VA 22030, USA
[3]Center for Molecular Engineering, George Mason University, Manassas, VA, 20110
[4] Department of Biomedical Sciences and Pathobiology, Virginia-Maryland College of Veterinary Medicine, Virginia Polytechnic Institute and State University, Blacksburg, VA 24061, USA
[5]Center for Emerging, Zoonotic, and Arthropod-borne Pathogens, Virginia Polytechnic Institute and State University, Blacksburg, VA 24061, USA
*E-mail: dklimov@gmu.edu

Restraints imposed on importin-α: The restraining sphere fixed in 3D space must continuously enclose the minNLS binding site of importin-α (impα). The minNLS binding site is defined in Methods (Sec. 2.7). To keep it within the sphere, we imposed Karplus-like restraints [1] to the protein. To define them, we need six points shown in Fig. S1. Three points P1-P3 are given by the atoms in the H3 helices from the impα ARM2-4 armadillo repeats, while three other points D1-D3 were chosen in space. One distance r and two angles (ϕ and θ) restrain the protein point P1 with respect to the sphere. Three other restraints associated with the angles Φ, Ψ, Θ control impα orientation. In Section "Structure of impα and its position with respect to the minNLS restraining sphere" we verify that the minNLS binding site of impα is enclosed within the restraining sphere.

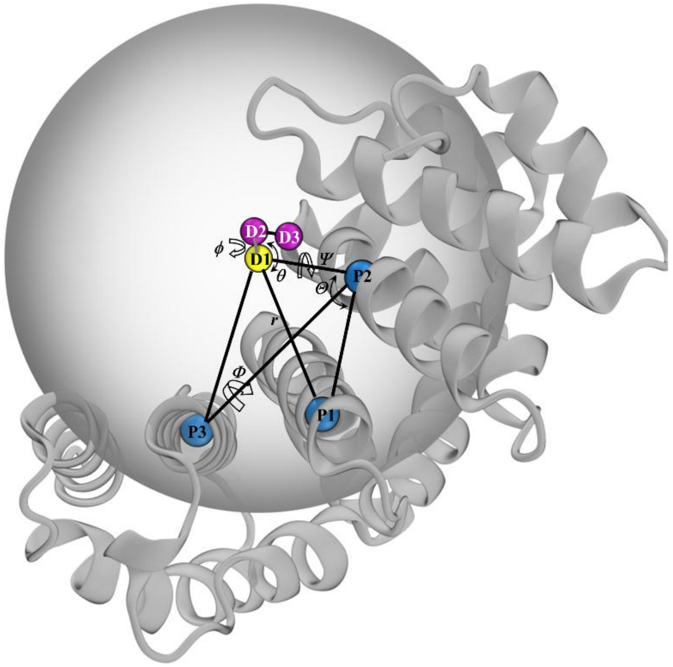

Figure S1 The design of Karplus-like restraints [1] applied to impα. The points P1-P3 and D1-D3 define the distance r (D1-P1), two angles ϕ (D3-D2-D1-P1) and θ (D2-D1-P1), three Euler-like angles Φ (D1-P3-P2-P1), Ψ (D2-D1-P2-P1), Θ (D1-P2-P1). The restraining sphere center is represented by yellow point D1.

FEP/REST setup: To evaluate the free energy of binding of KKPK peptide to impα, we used the absolute free energy perturbation method and replica exchange molecular dynamics with solute tempering (FEP/REST). The thermodynamic cycle in Fig. 1 involves three FEP/REST simulation stages, which first release the spherical restraint from the bound peptide (Restraint Bound, RB), then perform alchemical coupling of the bound peptide (Alchemical Bound, AB), and finally perform alchemical annihilation of the unbound peptide (Alchemical Unbound, AU). Details concerning RB, AB, and AU simulations are given in Methods. The RB simulations utilized R=15 conditions (λ_m,T_m), where λ_m is the scaling factor applied to the spring constant in the spherical restraint, T_m is the temperature, and $0 \leq m \leq R$-1. The RB conditions are given in Table S1.

Table S1: List of RB conditions.

m	**0**	**1**	**2**	**3**	**4**	**5**	**6**	**7**	**8**	**9**	**10**	**11**	**12**	**13**	**14**
λ_m	10.000	7.4347	5.3978	3.8112	2.6031	1.7079	1.0662	0.6250	0.3374	0.1627	0.0666	0.0211	0.0042	0.0003	0.0000
T_m	310	333	357	384	412	442	475	510	475	442	412	384	357	333	310

The AB simulations utilized R=40 conditions (λ_m,T_m), where λ_m is the alchemical coupling parameter, T_m is the temperature, and $0 \leq m \leq R$-1. The AB conditions are given in Table S2.

Table S2: List of AB conditions.

m	λ_m	T_m
**0**	0.0000	310.00
**1**	0.0186	316.78
**2**	0.0371	323.71
**3**	0.0557	330.80
**4**	0.0742	338.04
**5**	0.0928	345.43
**6**	0.1113	352.99
**7**	0.1299	360.72
**8**	0.1484	368.61
**9**	0.1670	376.67
**10**	0.1908	384.92
**11**	0.2146	393.34
**12**	0.2384	401.94
**13**	0.2621	410.74
**14**	0.2859	419.73
**15**	0.3097	428.91
**16**	0.3335	438.30
**17**	0.3573	447.89
**18**	0.3811	457.69
**19**	0.4049	467.70
**20**	0.4286	477.94
**21**	0.4524	488.39
**22**	0.4762	499.08
**23**	0.5000	510.00

m	λ_m	T_m
**24**	0.5590	494.38
**25**	0.6180	479.23
**26**	0.6770	464.55
**27**	0.7360	450.32
**28**	0.7950	436.52
**29**	0.8460	423.15
**30**	0.8850	410.18
**31**	0.9148	397.62
**32**	0.9376	385.44
**33**	0.9549	373.63
**34**	0.9682	362.18
**35**	0.9784	351.09
**36**	0.9861	340.33
**37**	0.9938	329.90
**38**	0.9976	319.80
**39**	1.0000	310.00

The AU simulations utilized R=32 conditions (λ_m,T_m), where λ_m is the alchemical coupling parameter, T_m is the temperature, and $0 \leq m \leq R$-1. The AU conditions are given in Table S3.

Table S3: List of AU conditions.

m	λ_m	T_m
**0**	0.0000	310.00
**1**	0.0200	318.69
**2**	0.0420	327.63
**3**	0.0640	336.82
**4**	0.0860	346.26
**5**	0.1100	355.98
**6**	0.1400	365.96
**7**	0.1700	376.22
**8**	0.2000	386.77
**9**	0.2300	397.62
**10**	0.2600	408.77
**11**	0.2900	420.23
**12**	0.3200	432.02
**13**	0.3500	444.13
**14**	0.3800	456.59
**15**	0.4100	469.39
**16**	0.4400	482.56
**17**	0.4700	496.09
**18**	0.5000	510.00
**19**	0.5750	490.84
**20**	0.6500	472.40
**21**	0.7250	454.65
**22**	0.7950	437.57
**23**	0.8550	421.13
**24**	0.9000	405.30
**25**	0.9294	390.08
**26**	0.9513	375.42
**27**	0.9675	361.32
**28**	0.9795	347.74
**29**	0.9885	334.68
**30**	0.9951	322.10
**31**	1.0000	310.00

FEP/REST performance: To assess FEP/REST technical performance, we followed the approach developed in our previous studies [2,3]. We first qualitatively assessed the random walks of replicas across the conditions. Fig. S2 demonstrates that RB, AB, and AU simulations produce a colorful random mosaic, in which replicas largely randomly walk across different conditions. These color mosaics do not reveal persistent trappings of replicas at any condition.

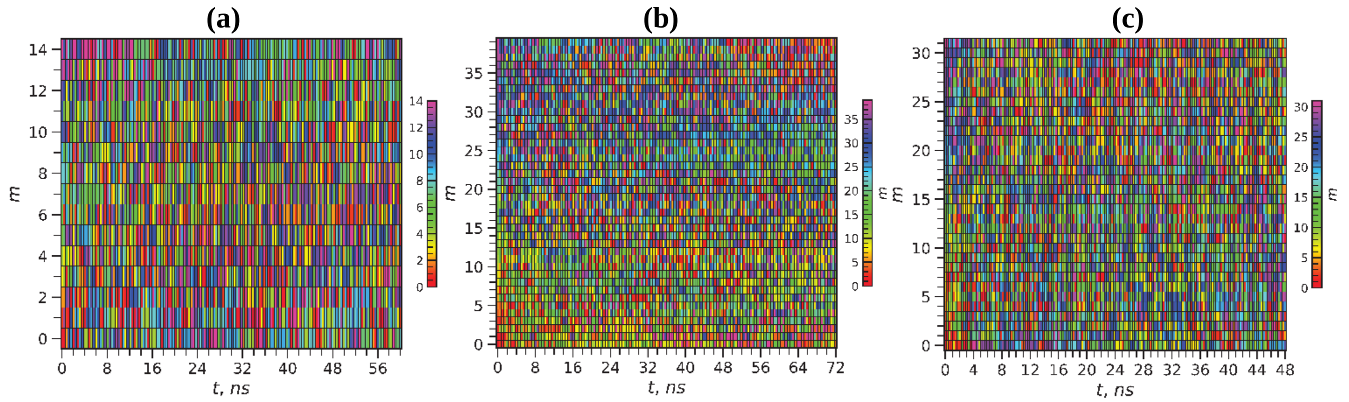

Figure S2 Walks of FEP/REST replicas across conditions m in the representative trajectories from RB (a), AB (b), and AU (c) simulations. The color scales assign replicas to conditions m at the start of the trajectory. Other FEP/REST trajectories exhibited similar behavior.

A quantitative probe of the distribution of replicas across the conditions is provided by the replica mixing parameter [4],

$$h(m) = 1 - \frac{\sqrt{\sum_{r=0}^{R-1} t_r^2}}{\sum_{r=0}^{R-1} t_r}, \qquad \text{(S1)}$$

where m is the FEP/REST condition and t_r is the time spent by replica r at the condition m. If FEP/REST simulation randomly mixes replicas across R conditions, $h(m)$ reaches the maximum optimum value $h_o = 1 - 1/R^{1/2}$. For RB, AB, and AU these optimum h_o values are 0.74, 0.84, and 0.82, respectively. Fig. S3 presents the mixing parameters $h(m)$ for RB, AB, and AU simulations and show that for all of them $h(m)$ approaches h_o. Indeed, the average h across all conditions are 0.71, 0.74, and 0.81. Although some deviation from h_o is noticeable for highly coupled conditions in AB simulations, we show below that it bears no apparent consequences on AB convergence. Finally, Fig. S4 presents the replica exchange rates $\alpha(m)$. Although there are some variations in $\alpha(m)$ across the conditions, the average exchange rates are 0.25, 0.26, and 0.18 for RB, AB, and AU simulations. All these rates are close to optimal [5]. Together Figs. S2-S4 implicate a good mixing of replicas across the conditions as prescribed by replica exchange formalism.

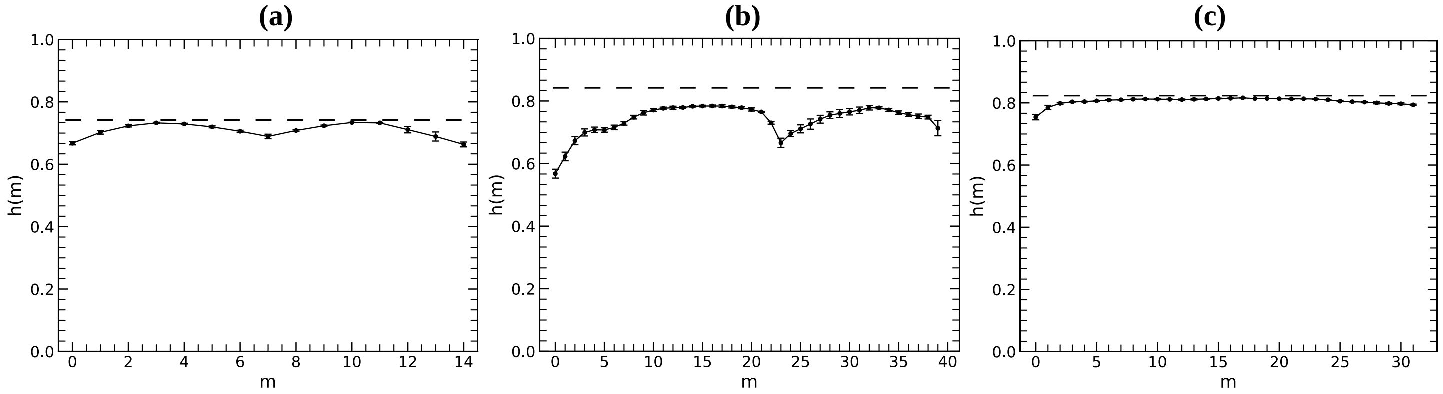

Figure S3 The replica mixing parameter $h(m)$ as a function of FEP/REST condition m for RB (a), AB (b), and AU (c) simulations. The maximum optimum values h_o is marked by dashed lines. The data is averaged across all FEP/REST trajectories.

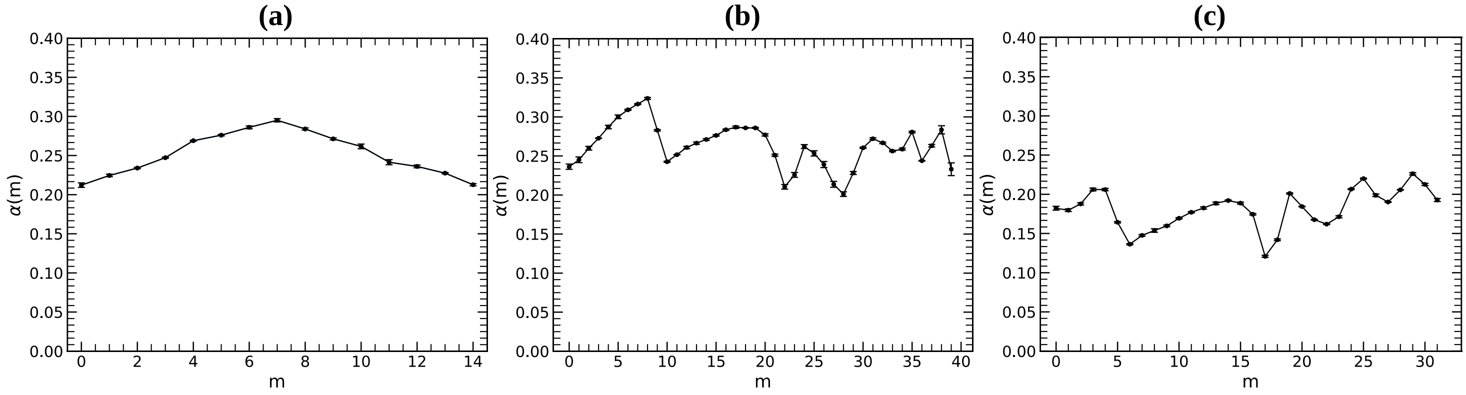

Figure S4 Replica exchange rates $\alpha(m)$ as a function of FEP/REST conditions m for RB (a), AB (b), and AU (c) simulations. The data is averaged across all FEP/REST trajectories.

Convergence of FEP/REST sampling: To evaluate the convergence of FEP/REST sampling, we devised two approaches. First, in RB and AB simulations we evaluated the root mean squared deviations (RMSD) of the

peptide-impα contact maps at the fully coupled RB and AB conditions m=0. To this end, we computed the contact maps $c(i,j;t,n)$ between minNLS j and impα i amino acids at FEP/REST time t in a trajectory n. Then, *cRMSD(t)* is defined as

$$cRMSD(t) = \left[\frac{1}{N_{tr}}\sum_n \frac{1}{N_c}\sum_{i,j}\left(c(i,j;t,n) - c^{ref}(i,j;t,n)\right)^2\right]^{\frac{1}{2}}, \qquad \text{(S2)}$$

where $c^{ref}(i,j;t,n)$ represents the contact map for the initial structure in a FEP/REST trajectory, N_{tr} is the number of FEP/REST trajectories, and N_c is the total number of possible peptide-impα contacts. Eq. (S2) tracks the formation of new or dissociation of existing binding contacts. Fig. S5a,b shows the respective *cRMSD(t)* for RB and AB simulations. It is seen that *cRMSD(t)* reaches approximate baseline after 20 ns in RB, but it reveals no appreciable equilibration process in AB simulations. To probe structural equilibration in AU simulations, we used the peptide radius of gyration $R_g(t)$ averaged over FEP/REST trajectories. Similar to AB Fig. S5c demonstrates no discernible equilibration process in AU simulations.

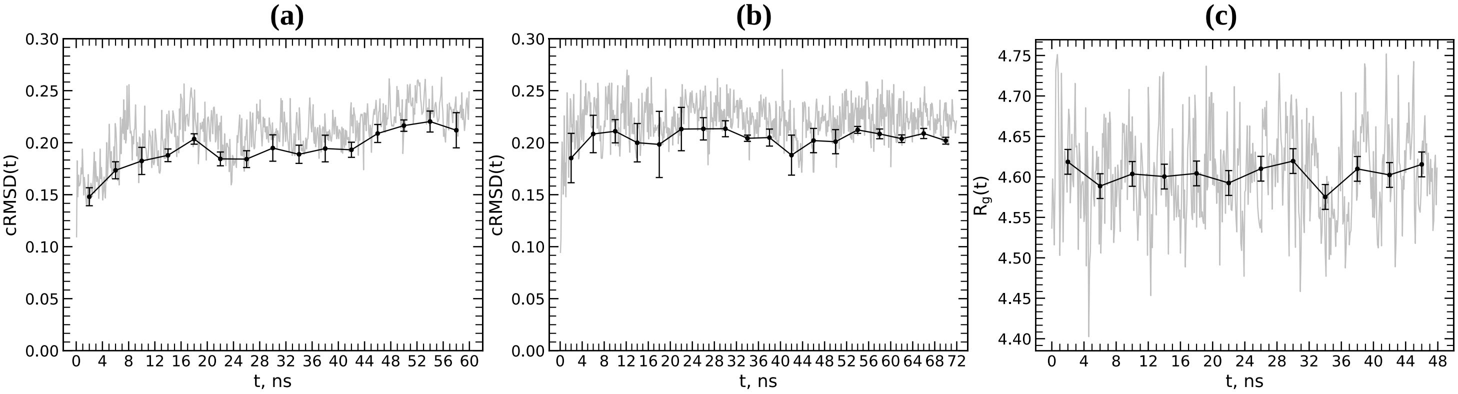

Figure S5 (a,b) The root mean squared deviations *cRMSD*(t) of binding contact maps computed as a function of FEP/REST time t for RB (a) and AB (b) simulations. (c) The peptide radius of gyration $R_g(t)$ as a function of FEP/REST time t in AU simulations. Data in grey is averaged over FEP/REST trajectories. Data in black is first computed using 4 ns batches and then averaged over FEP/REST trajectories.

In addition to testing the structural equilibration in Fig. S5, we assessed the convergence of FEP/REST simulations using the free energy changes occurring along the thermodynamic cycle in Fig. 1. Specifically, we computed the free energy change associated with the release of spherical restraint acting on the peptide KKPK bound to impα $\Delta G_{RB} = G_{BU} - G_{BR}$, where G_{BU} and G_{BR} are the free energies of the system with bound unrestrained and restrained peptides, respectively. Fig. S6a shows $\Delta G_{RB}(t)$ computed as a function of FEP/REST simulation time t. The plot reveals that $\Delta G_{RB}(t)$ has a small magnitude and fluctuates around the baseline. We further computed the entropic contributions to $\Delta G_{RB}(t)$ and found that at t > 32 ns it reaches a positive baseline. Positive entropic change indicates that the restraint release increases the peptide entropy as expected. Consequently, we assumed that RB simulations become equilibrated after t_{eq}=32 ns. Note that the free energy equilibration occurs after structural equilibration in Fig. S5a and therefore supersedes the latter. Next, we analyzed the convergence of the free energy change caused by recoupling of the peptide bound to impα $\Delta G_{AB} = G_{BC} - G_{BA}$, where G_{BC} and G_{BA} are the free energies of the system with bound coupled and annihilated peptides, respectively. As for RB simulations $\Delta G_{AB}(t)$ is computed as a function of FEP/REST simulation time t. In contrast to *cRMSD(t)* in Fig. S5b, $\Delta G_{AB}(k)$ in Fig. S6b reaches a baseline at $t_{eq} \approx 40$ ns. Finally, we considered the convergence of the free energy change caused by annihilation of the unbound peptide in water $\Delta G_{AU} = G_{UA} - G_{UC}$, where G_{UA} and G_{UC} are the free energies of the system with unbound annihilated and coupled peptides, respectively. As $R_g(t)$ in Fig. S5c the plot of $\Delta G_{AU}(t)$ in Fig. S6c as a function of t reveals no discernible equilibration. Merging this analysis together, we take as equilibrated the last 28 ns

in each RB trajectory, the last 32 ns in each AB trajectory, and the entire AU simulations. Since the numbers of RB, AB, and AU trajectories are five, three, and three, and they use R=15, 40, and 32 conditions, the total equilibrium sampling in these simulations amounts to 2.1, 3.84, and 4.608 μs, respectively. Binding energetics and conformational ensembles are computed using equilibrated sampling.

(a) **(b)** **(c)**

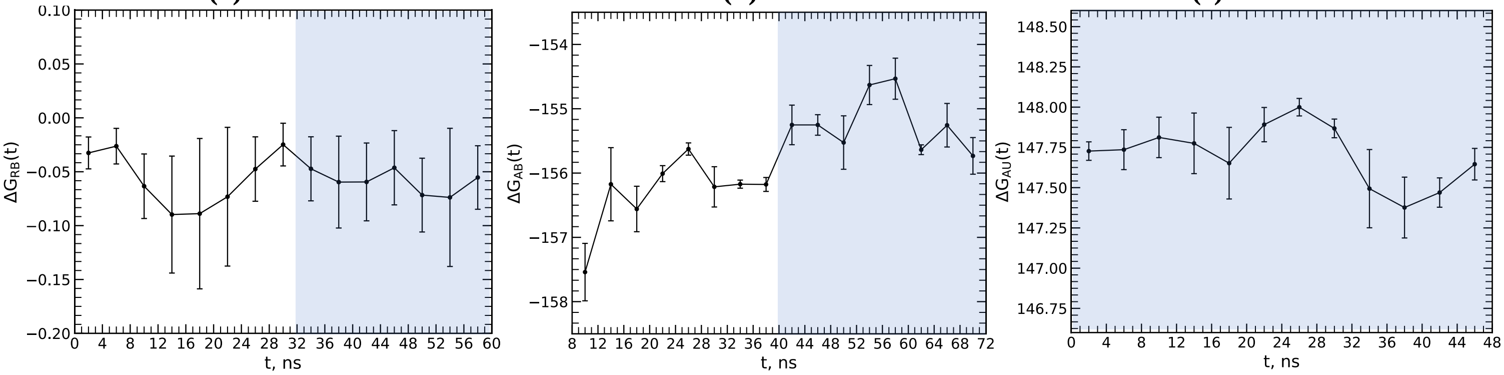

Figure S6 Convergence of free energy changes along the thermodynamic cycle: (a) The free energy change associated with the release of spherical restraint acting on the peptide KKPK bound to impα $\Delta G_{RB}(t)$. (b) The free energy change caused by coupling the peptide bound to impα $\Delta G_{AB}(t)$. (c) The free energy change caused by annihilation of the unbound peptide in water $\Delta G_{AU}(t)$. Free energies are computed as a function of FEP/REST simulation time t and averaged over 4 ns batch. The three panels establish the equilibration times t_{eq} in RB, AB, and AU simulations, which are equal or exceed the equilibration times suggested by structural properties in Fig. S5. The equilibrated portions of the simulations at $t > t_{eq}$ are shaded.

Structure of impα and its position with respect to the minNLS restraining sphere: Since impα is truncated at sequence position 211, lacks IBB domain, and remains conformationally unrestrained in the FEP/REST simulations, it is prudent to check that it maintains its native fold. To this end, we used the condition m=0 (λ_0=0,T_0=310K) from RB and AB simulations and computed the helical propensity $\langle H(i) \rangle$ for impα amino acids i. Fig. S7a compares the resulting *in silico* distribution of helical structure with that observed in the PDB 3VE6 structure. In addition, we computed the RMSD values between impα structures sampled in RB and AB simulations and the native PDB 3VE6 structure. To this end, we considered Cα atoms of impα minNLS binding site. The respective probability distribution $P(RMSD)$ is shown in Fig. S7b. The figure reveals a nearly perfect agreement between the two distributions of helical structure and generally low $RMSD$ values. The average $RMSD$ was found to be 1.8 Å. This result indicates that impα structure remains nearly native in our simulations.

(a) **(b)**

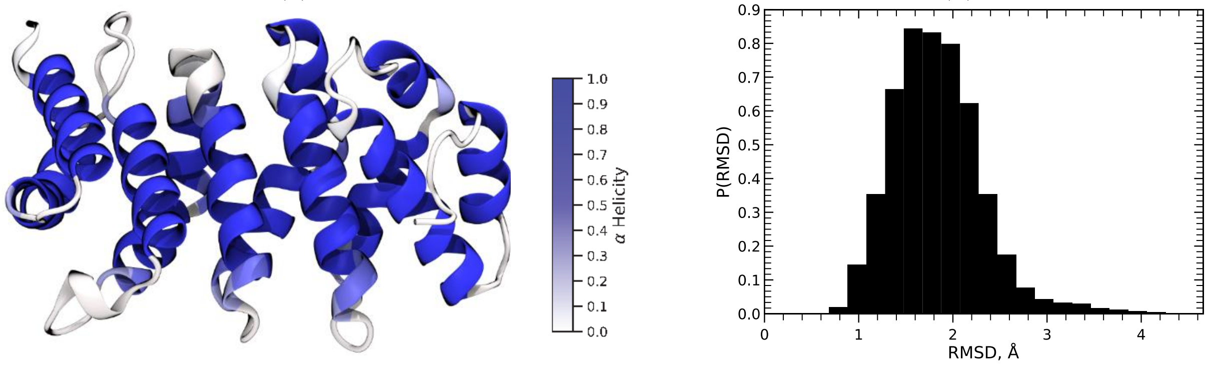

Figure S7 (a) The native structure of impα from 3VE6 PDB entry is shown in cartoon representation. The fraction of impα helical structure $\langle H(i) \rangle$ observed in FEP/REST simulations for amino acid i is represented by the shades of blue according to the scale. (b) Probability distribution $P(RMSD)$ of RMSD values comparing sampled impα structures vs 3VE6 structure. The figure demonstrates good agreement between the native and *in silico* structures.

Since we impose no restraints on impα conformations, its fluctuations may push the native minNLS binding site outside of the restraining sphere. To evaluate this possibility, we computed the probability P_o that any of 255 atoms belonging to the native minNLS binding site of impα occurs outside of the restraining sphere, i.e., their distance to the center of the sphere is greater than 18 Å. The resulting probability was found to be 0.16 and is primarily determined by impα Phe68. According to our previous REST simulations [2], the probability for KKPK to bind this amino acid is negligible (<0.001). If we exclude it, P_o is reduced to 0.03. The probability that the center of mass of a side chain belonging to the native minNLS binding site of impα occurs outside of the restraining sphere is lower being 0.02. It is worth noting that even if an impα atom exits the sphere, KKPK may still bind it as long as the peptide center of mass is enclosed in the sphere.

Conformational fluctuations of impα may cause the protein to occupy most of the restraining sphere volume leaving little space for the minNLS peptide. To evaluate this possibility, we used the number of water molecules N_w within the sphere in lieu of free volume estimate. Accordingly, we compared the probability distributions $P(N_w)$ computed using FEP/REST (RB+AB) and REST [2] simulations. The latter simulations have constrained the Cα atoms of amino acids belonging to the native minNLS binding site of impα to their native positions. Fig. S8 demonstrates that the sphere free volume in FEP/REST simulations has a wider distribution than in REST simulations with the respective standard deviations of 29 and 8, but the average values $< N_w >$ are very close (392 vs 399). These results suggest that due to unrestrained impα, the native minNLS binding site undergoes fluctuations, but, on average, the volume available for KKPK peptide within the sphere remains very close to the native value.

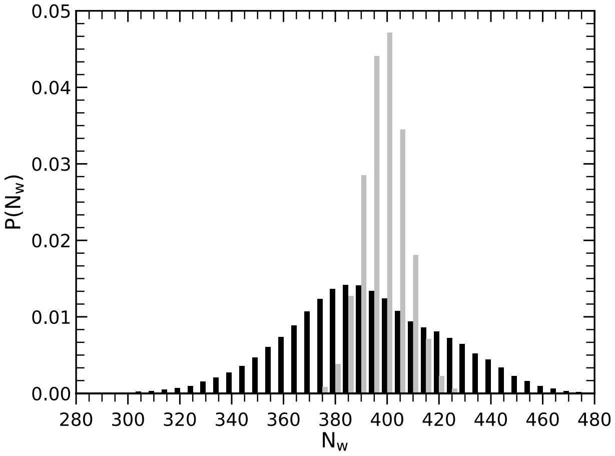

Figure S8 Probability distributions $P(N_w)$ of the number of water molecules N_w within the restraining sphere. Data in black and grey refer to FEP/REST and REST [2] simulations. These distributions estimate the free volume in the sphere available for minNLS.

Finally, due to a relatively small unit cell it is prudent to examine impα interactions across periodic boundaries. To this end, we computed the probability of forming any side chain contact between impα and any of its periodic images (see Methods). We found that in RB and AB simulations (condition m=0) these probabilities are 0.03 and 0.00. Thus, periodic boundary conditions artifacts appear insignificant.

Desolvation of lysine amino acids in minNLS peptide: We computed the release of water molecules from the first solvation shells (FSS) of lysine amino acids in the minNLS peptide caused by its binding to impα. Fig. S9 presents the corresponding probability distributions $P(N_w)$ of the numbers of FSS water molecules N_w in the bound and unbound states. The implications of these results are discussed in the main text.

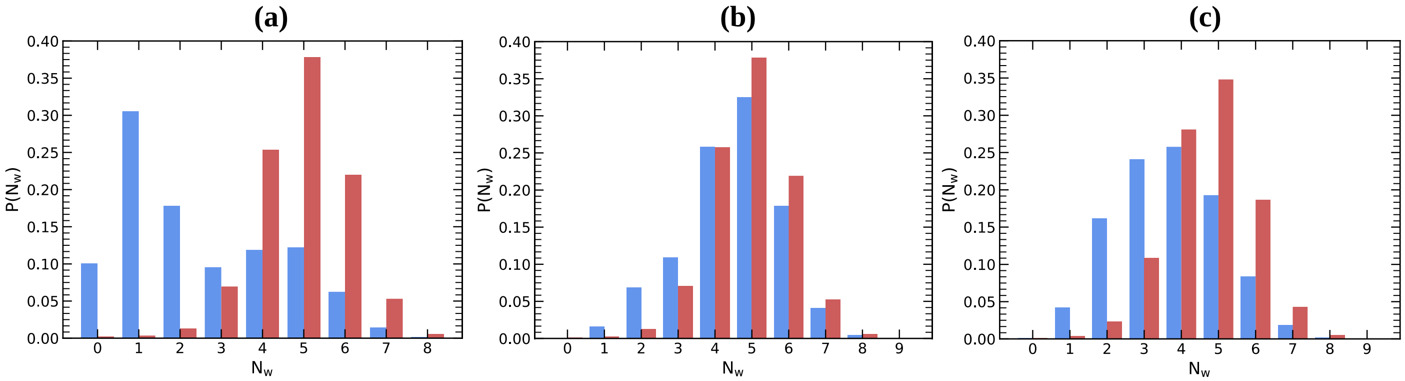

Figure S9 Probability distributions $P(N_w)$ of the numbers of water molecules in the FSS of lysine amino acids N_w in the bound (in blue) and unbound (in red) states. Panels (a)-(c) present $P(N_w)$ for Lys6, Lys7, and Lys9 of KKPK peptide, respectively.

Clustering the minNLS bound poses: Following the procedure described in Methods, we performed clustering of KKPK bound ensemble combining the AB and RB simulations (the conditions *m*=0). We identified 24 clusters collecting 50% of binding poses, which are presented in Table S4. The distribution of clusters is discussed in the main text.

Table S4 Bound clusters observed in FEP/REST simulations.

**Rank**	P_{cl}[a]	***RMSD***[b]**, Å**
**1**	0.08	2.0
**2**	0.05	9.7
**3**	0.04	12.1
**4**	0.04	11.0
**5**	0.04	8.0
**6**	0.03	3.9
**7**	0.03	9.0
**8**	0.02	7.3
**9**	0.02	10.2
**10**	0.02	2.9
**11**	0.02	8.6
**12**	0.02	4.2
**13**	0.01	11.2
**14**	0.01	4.6
**15**	0.01	11.6
**16**	0.01	9.6
**17**	0.01	9.1
**18**	0.01	10.9
**19**	0.01	8.9
**20**	0.01	10.1
**21**	0.01	9.9
**22**	0.01	7.7
**23**	0.01	9.4
**24**	0.01	8.6

[a] P_{cl} is the fraction of bound structures included in a cluster.

[b] RMSD is computed against the 3VE6 structure.

To directly compare the cluster distributions obtained from FEP/REST and REST [2] simulations, we computed the RMSD values between the centroids of respective clusters. FEP/REST and REST cluster distributions collect at least 50% of all sampled binding poses and represent the clusters with the highest populations P_{cl}. There are 24 such clusters in FEP/REST ensemble and 14 in REST simulations. The results are listed in Table S5 and discussed in the main text.

Table S5 Comparison of FEP/REST and REST cluster distributions.[a,b]

		**REST clusters**													
		**1**	**2**	**3**	**4**	**5**	**6**	**7**	**8**	**9**	**10**	**11**	**12**	**13**	**14**
**FEP/REST clusters**	**1**	5.4	10.3	9.7	8.9	3.3	6.2	5.4	1.2	15.1	7.0	8.0	8.5	8.0	4.4
	**2**	7.8	13.9	10.5	2.2	10.3	12.4	9.5	9.3	17.6	10.1	3.7	9.8	3.5	11.0
	**3**	10.4	8.1	7.3	13.0	10.9	8.8	9.6	11.6	14.6	12.3	12.8	7.4	12.8	10.3
	**4**	8.8	9.1	8.5	10.8	10.5	9.6	8.1	10.5	15.2	10.6	10.4	6.7	10.7	10.8
	**5**	3.0	9.3	9.7	9.0	7.9	7.8	1.8	6.8	12.7	9.0	8.3	3.9	8.1	8.4
	**6**	7.0	9.9	9.2	9.7	1.2	5.4	7.1	3.7	15.3	6.8	8.9	9.2	8.9	2.6
	**7**	7.6	13.7	10.9	2.5	9.7	11.9	9.2	8.7	17.8	10.0	2.4	9.9	3.9	10.3
	**8**	2.2	9.8	9.4	7.4	7.5	8.3	2.8	6.3	13.7	8.8	6.8	4.3	6.6	8.0
	**9**	8.8	14.5	11.2	2.5	10.9	13.0	10.3	9.9	18.1	10.4	3.9	10.8	4.2	11.6
	**10**	4.5	10.7	10.1	7.9	4.7	7.6	4.7	2.1	14.9	7.8	7.1	8.1	7.0	5.9
	**11**	6.1	12.9	10.2	1.9	9.3	11.2	8.0	8.2	16.9	9.2	2.7	8.5	2.2	9.9
	**12**	5.7	10.2	9.6	8.8	2.8	5.6	5.5	3.5	14.5	6.7	7.9	7.9	8.0	4.1
	**13**	8.2	8.3	9.7	12.6	10.7	9.4	6.9	10.4	13.6	11.4	12.3	6.1	12.1	11.0
	**14**	7.1	9.9	8.9	9.8	2.7	4.7	7.3	4.2	15.3	7.0	9.0	8.9	9.0	1.2
	**15**	7.8	9.0	9.8	11.9	11.0	9.8	6.7	10.8	13.2	11.7	11.7	5.3	11.4	11.4
	**16**	5.2	9.5	9.2	9.8	9.1	8.2	4.5	8.7	12.7	10.0	9.1	2.7	9.2	9.5
	**17**	4.4	9.2	9.1	9.1	8.6	7.6	4.1	8.0	12.9	9.4	8.4	2.3	8.4	8.6
	**18**	10.0	1.5	6.5	13.3	10.1	8.8	9.6	10.3	11.3	9.7	13.1	9.4	12.4	9.7
	**19**	4.4	9.2	9.6	10.1	8.6	7.3	2.8	7.7	12.5	9.2	9.4	3.5	9.4	8.8
	**20**	5.9	9.4	8.8	9.2	9.6	8.8	5.59	9.2	13.2	10.7	9.0	3.3	8.8	10.0
	**21**	9.4	13.4	10.5	8.7	9.5	10.1	10.2	9.8	20.4	10.7	8.4	9.5	8.7	9.0
	**22**	7.7	8.7	8.5	11.6	5.8	1.7	6.7	6.7	12.7	8.2	11.1	7.2	11.0	4.7
	**23**	8.7	10.8	8.0	8.5	8.9	8.8	9.1	9.2	17.7	10.4	8.6	8.2	8.8	8.3
	**24**	5.3	9.8	9.4	7.5	8.7	8.9	5.2	7.7	13.9	9.5	7.3	6.2	7.3	9.0

[a] The table presents the RMSD values in Å computed between the centroids of clusters.
[b] Yellow shaded RMSD values are < 3.0Å.

References

[1] Boresch, S., Tettinger, F., Leitgeb, M., and Karplus, M. (2003) Absolute Binding Free Energies: A Quantitative Approach for Their Calculation. *J. Phys. Chem. B* **107**, 9535-9551.

[2] Delfing, B. M., Laracuente, X. E., Olson, A., Foreman, K. W., Paige, M., Kehn-Hall, K., Lockhart, C., and Klimov, D. K. (2023) Binding of Viral Nuclear Localization Signal Peptides to Importin-α Nuclear Transport Protein. *Biophys. J.* **122**, 3476-3488.

[3] Delfing, B. M., Olson, A., Laracuente, X., Foreman, K. W., Paige, M., Kehn-Hall, K., Lockhart, C., and Klimov, D. K. (2023) Binding of Venezuelan Equine Encephalitis Virus Inhibitors to Importin-α Receptors Explored with All-Atom Replica Exchange Molecular Dynamics. *J. Phys. Chem.* B **127**, 3175–3186.

[4] Han, M. and Hansmann, U. H. E. (2011) Replica exchange molecular dynamics of the thermodynamics of fibril growth of Alzheimer's Aβ42 peptide. *J. Chem. Phys.* **135**, 065101.

[5] Denschlag, R., Lingenheil, M., and Tavan, P. (2009) Optimal temperature ladders in replica exchange simulations. *Chem. Phys. Lett.* **473**, 193-195.

[6] Qiao, B., Jiménez-Ángeles, F., Nguyen, T. D., and de la Cruz, M. O. (2019) Water follows polar and nonpolar protein surface domains. *Proc. Natl. Acad. Sci. USA* **116**, 19274–19281.

[7] Gilson, M. K., Given, J. A., Bush, B. L., and McCammon, J. A. (1997) The statistical-thermodynamic basis for computation of binding affinities: A critical review. *Biophys J*. **72**, 1047-1069.
